# Ionophore PBT2 as a novel approach to combat antibiotic-resistant *Helicobacter pylori*

**DOI:** 10.1128/mbio.01229-26

**Published:** 2026-06-15

**Authors:** Huiting Chen, Ibrahim M. El-Deeb, Chih-Ho Lai, Yu-Tsen Huang, Jessica Poole, Lauren E. Hartley-Tassell, Mark von Itzstein, Michael P. Jennings, Freda E.-C. Jen

**Affiliations:** 1 Institute for Biomedicine and Glycomics, Griffith University 265012, Gold Coast, Australia; 2 Department of Microbiology and Immunology, Graduate Institute of Biomedical Sciences, College of Medicine, Chang Gung University 210836, Taoyuan, Taiwan; NYU Langone Health, New York, New York, USA

**Keywords:** ionophore, PBT2, *Helicobacter pylori*, drug repurposing

## Abstract

*Helicobacter pylori* colonizes the gastric mucosa of around half of the world’s population and is a major cause of chronic gastritis, peptic ulcer disease, and gastric cancer. Current therapies are becoming increasingly ineffective due to the rapid spread of antibiotic resistance, creating an urgent need for new treatment options with distinct mechanisms of action. Drug repurposing offers a practical and cost-effective approach to address this gap. PBT2 is an 8-hydroxyquinoline derivative originally developed for the treatment of neurodegenerative diseases and has more recently been shown to possess antimicrobial activity. In this study, we demonstrate that PBT2 displays potent bactericidal activity against *H. pylori*, including multidrug-resistant clinical isolates. PBT2 rapidly killed *H. pylori in vitro* at low concentrations, with faster killing kinetics than commonly used antibiotics, and no resistance was detected after 30 days of continuous exposure. Importantly, PBT2 was effective in clearing an *H. pylori* infection in a murine model. Quantitative sequential window acquisition of all theoretical-mass spectrometry proteomic analysis revealed that PBT2 triggers broad disruption of essential bacterial processes, including global suppression of translation, impairment of iron-sulfur cluster assembly and respiration, dysregulation of metal homeostasis, and reduced abundance of virulence- and motility-associated proteins. We reported that PBT2 can act as a nickel ionophore, with Ni^2+^ being the highest-affinity ligand for PBT2 reported to date. Together, these findings suggest that PBT2 acts through a multifaceted, metal-dependent mode of action that limits the potential for emergence of resistance. Our work highlights PBT2 as a promising candidate for repurposing to treat multidrug-resistant *H. pylori* infections.

**IMPORTANCE:**

Antibiotic resistance is steadily reducing our ability to treat common bacterial infections, while the development of new antibiotics has slowed. *Helicobacter pylori* is a clear example of this growing problem, with treatment failures becoming more common worldwide. This study highlights the value of taking a different approach by repurposing existing drugs for new antibacterial uses. Rather than acting on a single bacterial target, the compound examined here disrupts multiple essential processes at once, reducing the probability of resistance developing.

## INTRODUCTION

*Helicobacter pylori* is a Gram-negative human pathogen that colonizes the gastric mucosa of half the global population (1). While infections remain asymptomatic in most individuals, persistent colonization of the stomach by *H. pylori* can lead to chronic gastritis, peptic ulcer, and is closely linked to mucosa-associated lymphoid tissue lymphoma and gastric cancer (GC) (2–4). GC remains the fifth most common cancer and the fourth leading cause of cancer mortality worldwide (5). Approximately 3% of infected patients develop GC, and 90% of non-cardia gastric cancer is caused by *H. pylori* infection (6, 7). Consequently, *H. pylori* is classified as a group I carcinogen by the World Health Organization (8). The world prevalence has been in a gradual decline from over 50% –55% to 43% in adults over the last few decades due to improved hygiene conditions (9, 10). Although the overall prevalence has been declining, infection remains high in children and adolescents (35.1%) (9). The current efficacy of standard triple or quadruple therapies is significantly diminishing due to the rapid emergence of antibiotic resistance (11, 12). Clarithromycin resistance rates exceed 15% in many regions, while the metronidazole resistance rate is even more prevalent, reaching over 50% in most regions and peaking at 91% in Africa (12). Levofloxacin resistance is also common, with most regions reporting rates above 20% (12). Consequently, first-line and rescue regimens recommended worldwide fail in 10%–30% of patients, reflecting declining eradication success rates over the past two decades (13). Despite the growing burden of resistance, no new antibiotics or efficient vaccines have been launched for *H. pylori* in recent years. Consequently, there is an urgent need to explore alternative treatments with novel mechanisms of action. Apart from scientific barriers, the high risk of failure, escalating development costs, and prolonged timelines have contributed to a significant decline in investment in the antibiotic development pipeline (14, 15).

Drug repurposing offers a promising, time- and cost-effective approach (16). PBT2 is an 8-hydroxyquinoline derivative originally developed as a human drug candidate for treating Alzheimer’s disease (17, 18) and later studied for Huntington’s disease (19). Its metal-binding properties enable it to chelate and redistribute transition metals such as zinc and copper that contribute to neurodegenerative pathology in the brain. PBT2 has recently gained attention for its antimicrobial properties as an antibacterial adjuvant or potentiator. PBT2 and zinc exhibit antibacterial activity against both gram-positive and gram-negative bacteria (20, 21). Further research has shown that PBT2, when combined with zinc and conventional antibiotics, can reverse resistance phenotypes in several bacterial species through metal-mediated disruption of cellular processes (22–28). Our recent studies have established that PBT2 alone, without zinc or additional antibiotics, is a potent inhibitor of bacterial pathogens such as *Neisseria meningitidis* and *Neisseria gonorrhoeae*, with low concentrations sufficient to achieve effective treatment (29). These data prompted investigation into whether PBT2 alone may also have therapeutic potential against *H. pylori*.

## RESULTS

### PBT2 has a potent bactericidal activity against *H. pylori in vitro*

To evaluate the potential antimicrobial effect of PBT2 on *H. pylori*, minimum inhibitory concentration (MIC) testing was performed using broth microdilution in brain and heart infusion (BHI) broth with 10% fetal bovine serum under microaerophilic conditions (30) and also by agar diffusion, as recommended by the Clinical and Laboratory Standards Institute (CLSI) (31). Reference laboratory strains ATCC 43526, ATCC 700392 (26695) (32), J99 (33), SS1 (34), and two multidrug-resistant clinical isolates CH426 and CH428 (35) were tested. PBT2 was synthesized following procedures previously optimized and validated by our group (22). PBT2 inhibited growth at 0.625 mg/L–5 mg/L across all isolates (see Table 1). Importantly, resistance to conventional antibiotics did not alter susceptibility to PBT2. The MDR strains CH426 and CH428, which were previously shown to be resistant to metronidazole, clarithromycin, and amoxicillin (35), remained susceptible to PBT2. These results indicate that the existing resistant mechanisms in CH426 and CH428 did not impact PBT2 efficacy.

**TABLE 1:** MICs of PBT2 against *H. pylori* reference strains, mouse adaptable strain, and MDR isolates^*a*^^,^^*b*^

Strain	PBT2 (mg/L) agar diffusion	PBT2 (mg/L) broth dilution	Tetracycline (mg/L) broth dilution	Amoxicillin (mg/L) broth dilution
ATCC 43526	2.5	1.25	0.25	0.125
ATCC 700392	5	2.5	0.25	0.0625
CH426^*c*^	5	0.625	0.5	>256 (E-test)^*c*^
CH428^*c*^	2.5	2.5	0.25	>256 (E-test)^*c*^
J99^*d*^	2.5	0.625	0.125	0.015
SS1^*d*^	2.5	1.25	0.125	0.125

^*a*^ CLSI quality control (QC) ranges: tetracycline: 0.12 mg/L–1 mg/L, amoxicillin: 0.015 mg/L–0.12 mg/L.

^*b*^ ATCC 43526 and ATCC 700392 are reference strains used as the CLSI standard for antimicrobial susceptibility testing.

^*c*^ CH426 and CH428 are clinical strains that are resistant to amoxicillin, metronidazole, and clarithromycin reported in Nguyen et al. (35).

^*d*^ J99 and SS1 are clinical isolates that have been adapted for use in mouse models.

### Antibacterial activity of PBT2 against *H. pylori*

**Fig 1:**
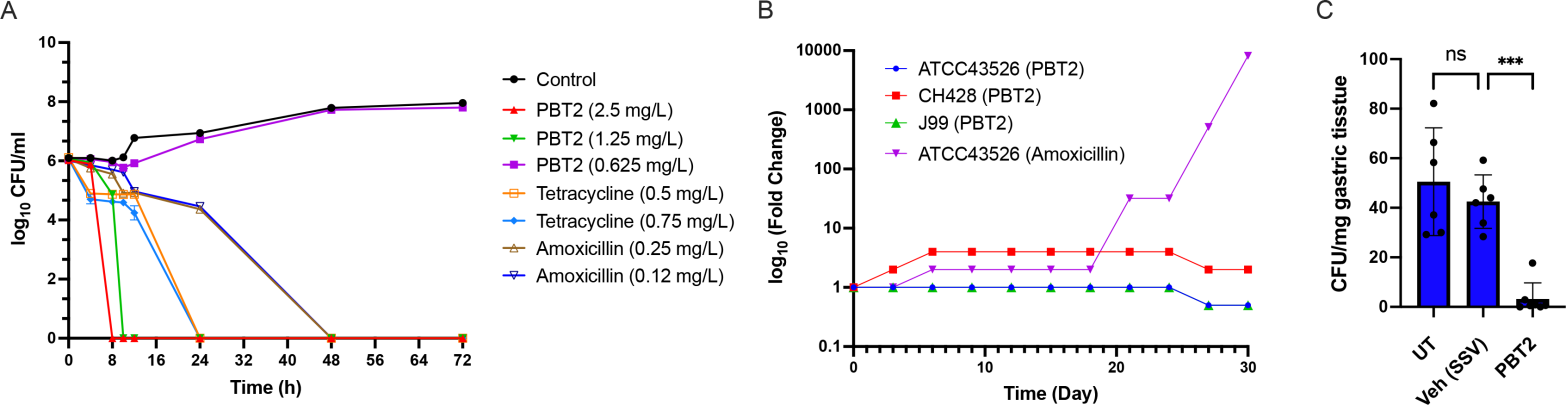
The effect of PBT2 on *H. pylori*. (**A**) Time-kill kinetics of PBT2 against *H. pylori* ATCC 43526. Bactericidal curves are shown for the control culture (no drug) and cultures treated with PBT2 at 2× MIC (2.5 mg/L), 1× MIC (1.25 mg/L), and 0.5× MIC (0.625 mg/L) in BHI broth + 10% fetal bovine serum. Tetracycline and amoxicillin were included as controls. Data represent means ± standard deviations (SDs) of three biological replicates. (**B**) Resistance development to PBT2 in *H. pylori* ATCC 43526, CH428, and J99. The fold change was calculated by normalizing the MIC acquired for a serial passaging subculture to the original MIC. Amoxicillin was included as a control for ATCC 43526. Each data point represents three biological replicates. (**C**) PBT2 eliminates *H. pylori* in a murine infection model. CFUs were counted from gastric tissue of BALB/c infected with ATCC 700392. UT, untreated; Veh, solvent vehicle (SSV), which consisted of 0.9% wt/vol NaCl, 0.5% wt/vol sodium carboxymethylcellulose, 0.5% vol/vol benzyl alcohol, and 0.4% vol/vol Tween-80. Untreated; *** *P*  <  0.0001, vehicle versus PBT2 treatment; ns, non-significant; UT versus Veh.

To determine whether PBT2 exhibits bactericidal or bacteriostatic activity and to define its killing kinetics against *H. pylori*, time-kill assays were performed using the reference strain ATCC 43526. Mid-log-phase cultures were exposed to PBT2 at 2× MIC (2.5 mg/L), 1× MIC (1.25 mg/L), and 0.5× MIC (0.625 mg/L), and viable counts were quantified over 72 h. Amoxicillin and tetracycline were included at 2× MIC (0.25 mg/L and 0.5 mg/L, respectively) as comparator antibiotics. PBT2 demonstrated rapid, concentration-dependent bactericidal activity (Fig. 1A). At 2× MIC, complete killing was achieved within 8 h, while exposure to 1× MIC resulted in complete killing by 10 h, with no recoverable colonies detected at 72 h for either concentration. In contrast, treatment with 0.5× MIC produced an initial reduction in colony-forming unit (CFU) counts, followed by regrowth after 12 h, although final CFU/mL values remained lower than those of untreated controls throughout the assay. Both amoxicillin and tetracycline exhibited substantially slower killing kinetics at 2× MIC, requiring more than 24 h to achieve comparable reductions in viable counts (Fig. 1A). Collectively, these results establish that PBT2 mediates rapid and sustained bactericidal activity against *H. pylori* compared with conventional antibiotics under equivalent conditions.

Because *H. pylori* is a host-adapted, human-specific pathogen capable of establishing lifelong colonization, it is under constant selective pressure from host immunity and, latterly, therapeutic intervention. This creates an environment where resistance can readily emerge and persist within the gastric niche. Therefore, it was essential to evaluate the potential for resistance development under sustained PBT2 exposure. To evaluate the potential for resistance development, *H. pylori* was serially passaged for 30 days in sub-inhibitory concentrations of PBT2, with MIC determination every third passage. PBT2 MICs remained stable throughout the 30-cycle study, indicating no detectable progressive adaptation under prolonged sub-inhibitory exposure, while parallel cultures exposed to amoxicillin developed >30-fold increases in MIC (Fig. 1B).

### Evaluation of PBT2 activity in a murine model of *H. pylori* infection

To assess whether the potent *in vitro* activity of PBT2 translated to an *in vivo* setting, its efficacy was evaluated in a murine gastric infection model of *H. pylori* as previously described (34, 36–38). BALB/c mice were infected by repeated intragastric inoculation and subsequently treated with PBT2 administered orally twice daily for 3 days, following a previously established protocol for PBT2 oral treatment in mice (23). Control groups received either the Standard Suspension Vehicle (SSV) or no treatment. Control experiments confirmed that the solvent vehicle components do not contribute to bactericidal activity under the conditions tested. Gastric colonization was quantified by culture of homogenized gastric tissue and expressed as CFU per mg of tissue. Colonization levels in this model were relatively low, which is consistent with previous reports using BALB/c mice infected with non–mouse-adapted *H. pylori* strains (34, 36–38). Mice receiving PBT2 exhibited a significant reduction in gastric *H. pylori* burden compared with both untreated and vehicle-treated controls (Fig. 1C). In the PBT2-treated group, bacterial loads were reduced to near or below the limit of detection in most animals. In contrast, untreated mice and those receiving vehicle alone remained colonized, with no significant difference observed between these two control groups. Statistical analysis confirmed a significant reduction in CFU/mg gastric tissue in the PBT2-treated group relative to vehicle-treated controls (***P < 0.0001), whereas no significant difference was detected between untreated and vehicle-treated animals (ns). These results demonstrate that short-course oral administration of PBT2 significantly reduces *H. pylori* gastric colonization *in vivo*, supporting its antimicrobial activity in a murine infection model.

### Global proteomic changes in *H. pylori* following PBT2 exposure

To elucidate the molecular basis of PBT2 antimicrobial activity, we performed Sequential window acquisition of all theoretical-mass spectrometry (SWATH-MS)-based quantitative proteomic profiling of *H. pylori* ATCC 43526. Time-kill data were used to guide selection of sampling points corresponding to distinct physiological states. Cultures were exposed to PBT2 at 1× MIC for 8 h (T8), representing terminal bactericidal stress, or at 0.5× MIC for 10 h (T10) and 12 h (T12), corresponding to peak and sustained sublethal responses, respectively. Proteins were extracted, trypsin-digested, and analyzed by LC-MS/MS. These time points provided a framework for comparing proteomic responses associated with terminal stress and sustained sublethal exposure.

**Fig 2:**
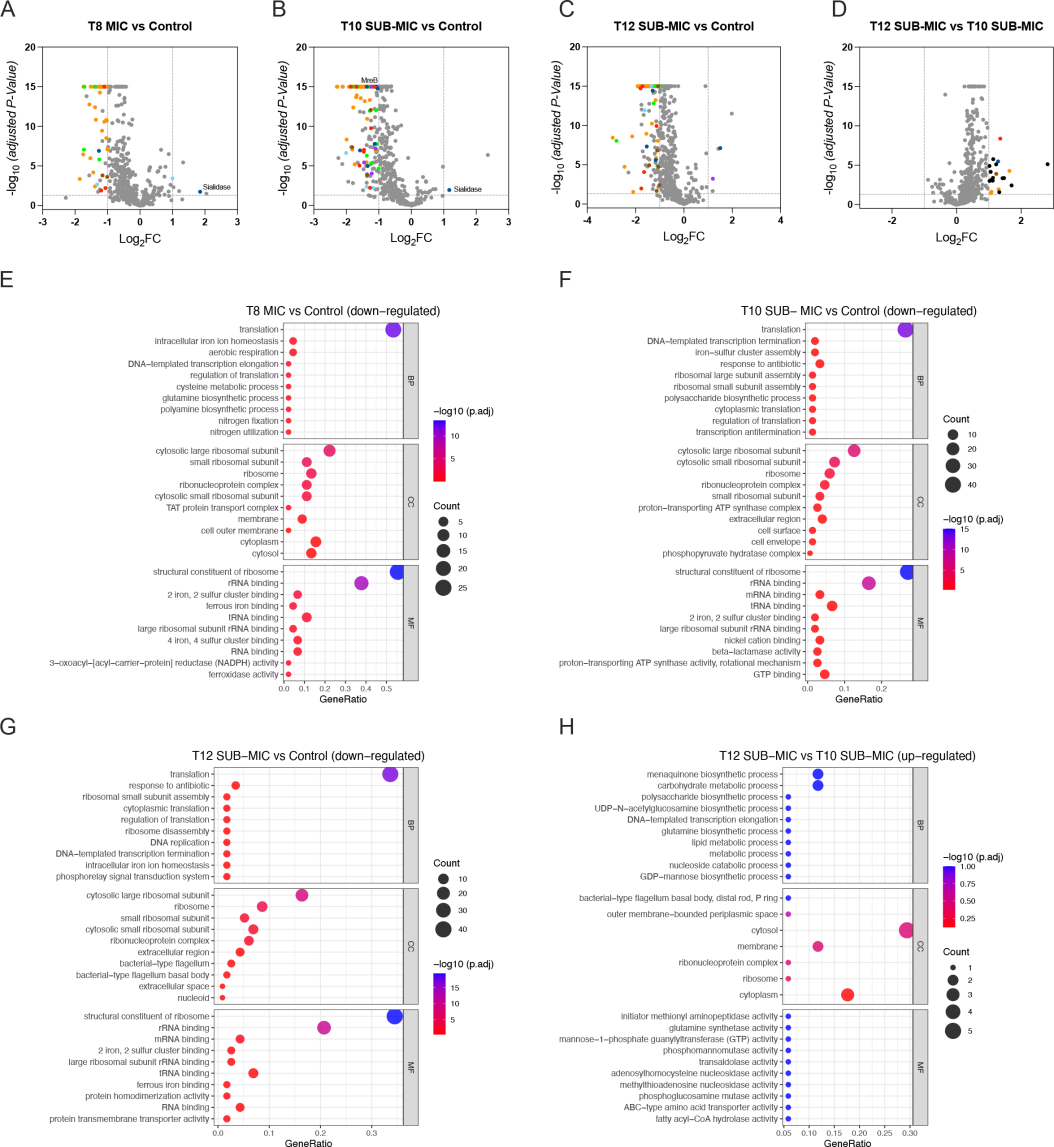
Proteomic responses of *H. pylori* ATCC 43526 to PBT2 treatment. (**A–D**) Volcano plots showing protein abundance changes across treatment groups in ATCC 43526 *H. pylori* at different time points. (**A**) PBT2 treatment at 1× MIC (1.25 mg/L) for 8 h vs untreated control. (**B**) PBT2 treatment at 0.5× MIC (0.625 mg/L) for 10 h vs untreated control. (**C**) PBT2 treatment at 0.5× MIC (0.625 mg/L) for 12 h vs untreated control. (**D**) PBT2 treatment at 0.5 × MIC (0.625 mg/L) for 12 h vs 10 h. Differentially abundant proteins were defined using the threshold adj. *P* < 0.05, log₂FC > 1 (dashed line). Proteins of interest are highlighted: orange, translation machinery; green, Fe-S cluster proteins; blue, respiratory chain components; red, metal acquisition and storage; navy, outer membrane adhesins/proteins; purple, Cag proteins; brown, motility and chemotaxis proteins. (**E–H**) Gene Ontology (GO) enrichment of differentially abundant proteins in ATCC 43526 *H. pylori* under PBT2 treatment. The *y*-axis shows enriched GO terms (BP, biological process; MF, molecular function; CC, cellular component), and the *x*-axis represents the gene ratio (observed/background proteins). Bubble size represents the number of proteins mapped to each category, and bubble color corresponds to −log10 adjusted *P-*value. (**E**) PBT2 treatment at 1× MIC (1.25 mg/L) for 8 h vs untreated control. Differentially downregulated proteins were analyzed. (**F**) PBT2 treatment at 0.5× MIC (0.625 mg/L) for 10 h vs untreated control. Differentially downregulated proteins were analyzed. (**G**) PBT2 treatment at 0.5× MIC (0.625 mg/L) for 12 h vs untreated control. Differentially downregulated proteins were analyzed. (**H**) PBT2 treatment at 0.5× MIC (0.625 mg/L) for 12 h vs 10 h. Differentially upregulated proteins were analyzed.

Across all conditions and replicates, a total of 839 non-redundant *H. pylori* proteins were identified (false discovery rate < 1%) and used as the background for differential abundance analysis. Comparative analysis revealed widespread changes in protein abundance, with between 20 and 172 proteins exhibiting at least twofold differential abundance across conditions (adjusted *P* < 0.05; Fig. 2A through D; Table S1). In all drug-control comparisons, the dominant pattern was a reduction in protein abundance (Fig. 2A through C), whereas the comparison between 12 h and 10 h sub-MIC exposure showed a bias toward increased protein abundance, indicating a time-dependent adaptive shift under prolonged sublethal stress (Fig. 2D). Selected proteins showing significant and biologically relevant changes in abundance across conditions are summarized in Table 2, highlighting key alterations in virulence, metal homeostasis, Fe-S cluster biology, and nickel metabolism.

**TABLE 2:** Representative differentially abundant proteins in *H. pylori* under bactericidal and sublethal PBT2 exposure conditions

Condition	Protein	Function	Category	Fold change^*b*^	Adjusted *P*-value^*a*^
T8, 1× MIC (1.25 mg/L)	Sialidase	Host interaction	Virulence	3.61 ↑	0.0179
	NifU	Fe-S cluster assembly	Fe-S cluster biology	3.27 ↓	0
	FecA	Iron transporter	Metal homeostasis	2.29 ↓	0.0128
	CopD	Copper resistance protein	Metal homeostasis	2.09 ↓	0.0061
T10, 0.5× MIC (0.625 mg/L)	Sialidase	Host interaction	Virulence	2.25 ↑	0.0111
	FrpB	Iron-regulated outer membrane protein	Metal homeostasis	3.21 ↓	0
	NifU	Fe-S cluster assembly	Fe-S cluster biology	3.18 ↓	0
	Omp32	Outer membrane protein	Virulence	2.99 ↓	1.1 × 10⁻⁷
	CopD	Copper resistance protein	Metal homeostasis	2.75 ↓	1.63 × 10⁻⁷
	UreF	Urease accessory protein	Nickel metabolism	2.74 ↓	9.58 × 10⁻⁸
	HypB	Urease maturation factor	Nickel metabolism	2.49 ↓	0
	UreG	Urease accessory protein	Nickel metabolism	2.18 ↓	0
T12, 0.5× MIC (0.625 mg/L)	Cag19	Cag pathogenicity island protein	Virulence/host interaction	2.29 ↑	0.000606
	FrpB	Iron-regulated outer membrane protein	Metal homeostasis	3.28 ↓	0
	CopD	Copper resistance protein	Metal homeostasis	3.13 ↓	8.35 × 10⁻⁵
	Omp6	Outer membrane protein	Virulence	2.90 ↓	4.81 × 10⁻⁸
	NifU	Fe-S cluster assembly	Fe-S cluster biology	2.27 ↓	0
T12 vs T10, 0.5× MIC	DsbC	Protein folding/stress adaptation	Stress response	7.08 ↑	7.61 × 10⁻⁶
	UreF	Urease maturation factor	Nickel metabolism	2.56 ↑	4.19 × 10⁻⁹
	Omp32	Outer membrane protein	Virulence	2.44 ↑	3.32 × 10⁻⁶

^*a*^ *P*-values below 1 × 10⁻¹⁵ were reported as zero by the analysis software.

^*b*^ Arrows represent proteins up- or downregulated by PBT2.

Gene Ontology enrichment analysis demonstrated that proteins reduced in abundance were strongly associated with translation and ribosome structure, including 30S and 50S ribosomal subunits and rRNA binding (Fig. 2E through H). Proteins involved in Fe-S cluster binding and assembly were also significantly enriched among those decreased, along with components of the respiratory chain. In addition, proteins involved in metal acquisition, transport, and storage—encompassing iron, copper, zinc, and nickel-associated systems such as urease maturation factor HypB, urease accessory proteins UreF and UreG—were broadly reduced. Together, these changes indicate disruption of core processes required for energy production, redox balance, and metal homeostasis during PBT2 exposure. Virulence-associated proteins were also suppressed, including multiple outer membrane adhesins and porins, components of the Cag pathogenicity island, proteins involved in motility and chemotaxis, and the cell shape determinant MreB (Fig. 2E through H; Table S1). In contrast, sialidase was increased in abundance under PBT2 treatment, suggesting a distinct response among adhesion-associated factors.

At later sub-MIC time points, selective increases were observed in proteins involved in quinone biosynthesis, lipoproteins, and urease accessory proteins, consistent with partial reactivation of metabolic and envelope-associated processes. However, ribosomal proteins remained suppressed under these conditions. Collectively, these proteomic data indicate that PBT2 exposure results in coordinated suppression of protein synthesis, respiration, virulence-associated systems, and metal homeostasis in *H. pylori*, while permitting limited adaptive responses during sustained sublethal stress.

### Isothermal titration calorimetry (ITC) analysis of PBT2-metal interactions

SWATH-MS proteomic analysis revealed broad suppression of proteins involved in metal homeostasis following PBT2 exposure, including proteins associated with iron, copper, zinc, and nickel utilization. In *H. pylori*, nickel is of particular importance, as it is an essential cofactor for urease and [NiFe]-hydrogenase, enzymes required for gastric colonization and survival in the acidic environment of the stomach (39). Changes in the abundance of urease accessory proteins were observed under PBT2 treatment, suggesting perturbation of nickel-dependent processes. These findings prompted further investigation into whether PBT2 can directly interact with nickel ions. To address this, we performed ITC to quantify the binding affinity and stoichiometry of PBT2 interactions with Ni^2+^, alongside Zn^2+^ and Cu^2+^, which have been previously reported to interact with PBT2.

To determine the binding affinity and stoichiometry of PBT2 interactions with divalent metals, ITC was conducted. Titration of Zn^2+^ into PBT2 produced well-defined heat changes with a dissociation constant (K_D_) value of 2.80 ± 1.14 µM (2:1 molar ratio) (Fig. S1A; Table 3). The binding stoichiometry and affinity of PBT2 to zinc were in good agreement with previous reports (20). Previous structural studies reported Cu^2+^ can form complexes with PBT2 in either 2:1 or 1:1 stoichiometries (40–42). In our data, we showed a 1:1 interaction with a moderate affinity of 2.64 ± 1.27 µM under the tested condition (Fig. S1B; Table 3). Nickel binding to PBT2 has not been previously reported. Our data revealed that Ni^2+^ binds to PBT2 with a stoichiometry best fitted to a 2:1 model and an apparent K_D_ of 378 ± 72.1 nM (Fig. S1C; Table 3), indicating that Ni^2+^ is the highest-affinity ligand for PBT2 reported to date. Mg^2+^ titration was included as a negative control. No detectable interaction was detected between Mg^2+^ and PBT2 under the conditions tested (Fig. S1D).

**TABLE 3:** Thermodynamic properties of PBT2 to different metals using ITC^*a*^

Metal	K_D_ (M)	*n*	ΔH (kJ/mol)	ΔS (kJ/mol)
ZnSO₄·7H₂O	2.80 ± 1.14E-06	0.428 ± 0.02	−20.20 ± 4.57	41.69 ± 18.02
CuSO_4_·5H_2_O	2.64 ± 1.27E-06	0.901 ± 0.01	−19.56 ± 1.27	44.51 ± 6.35
NiSO_4_·6H_2_O	3.78 ± 0.72E-07	0.578 ± 0.03	−6.21 ± 2.40	103.04 ± 6.56
MgSO_4_	NCDI	NCDI	NCDI	NCDI

^*a*^ NCDI, no calorimetrically detectable interaction. The data were calculated from triplicates and presented as mean ± SD.

### PBT2 disrupts intracellular metal homeostasis in *H. pylori*

**Fig 3:**
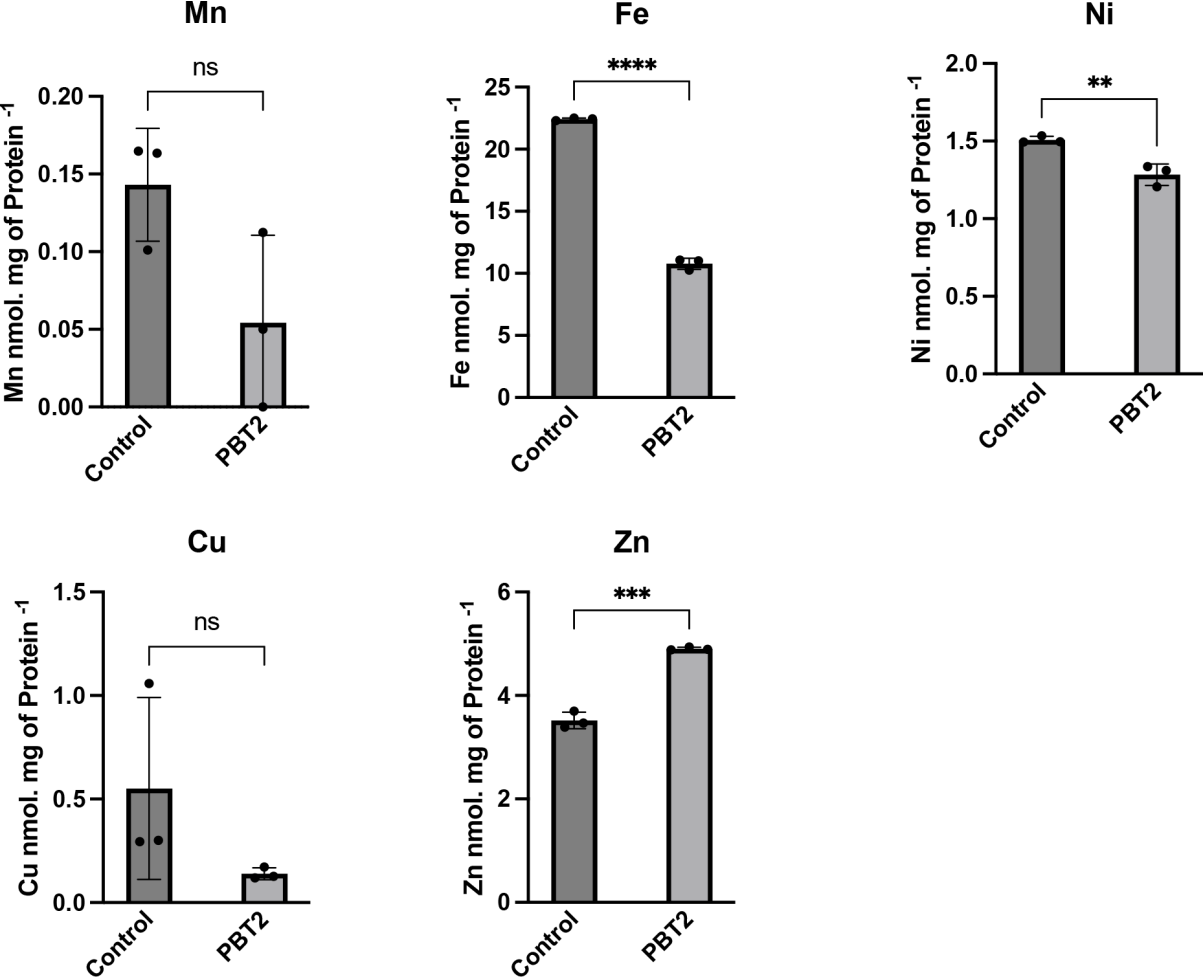
Intracellular metal concentrations as determined by ICP-MS for *H. pylori*. (Error bars indicate standard error of the mean from at least three biological replicates; **, *P* < 0.005; ***, *P* < 0.001; ****, *P* < 0.0001, unpaired two-tailed *t*-test).

To determine whether PBT2 perturbs metal homeostasis in *H. pylori*, we quantified intracellular metal content following short-term exposure using inductively coupled plasma mass spectrometry (ICP-MS). Mid-log phase cultures of *H. pylori* ATCC 43526 were treated with PBT2 at 1× MIC (1.25 mg/L) for 1 h under microaerophilic conditions, and cellular metal levels were normalized to total protein content. PBT2 treatment resulted in selective and significant alterations in intracellular metal composition (Fig. 3). Iron levels were markedly reduced compared to untreated controls (****P < 0.0001), indicating substantial disruption of iron homeostasis. Nickel levels were also significantly decreased (**P < 0.01), consistent with perturbation of nickel-dependent processes. In contrast, zinc levels were significantly increased following PBT2 exposure (***P < 0.001). No significant changes were observed for manganese or copper under the same conditions.

The observed pattern of metal redistribution characterized by depletion of iron and nickel alongside accumulation of zinc indicates that PBT2 induces selective dysregulation of intracellular metal pools rather than a global loss of cellular ions. These findings are consistent with the ionophoric properties of PBT2 and support a mechanism involving disruption of metal homeostasis in *H. pylori*.

### Effect of PBT2 on *H. pylori* urease activity

**Fig 4:**
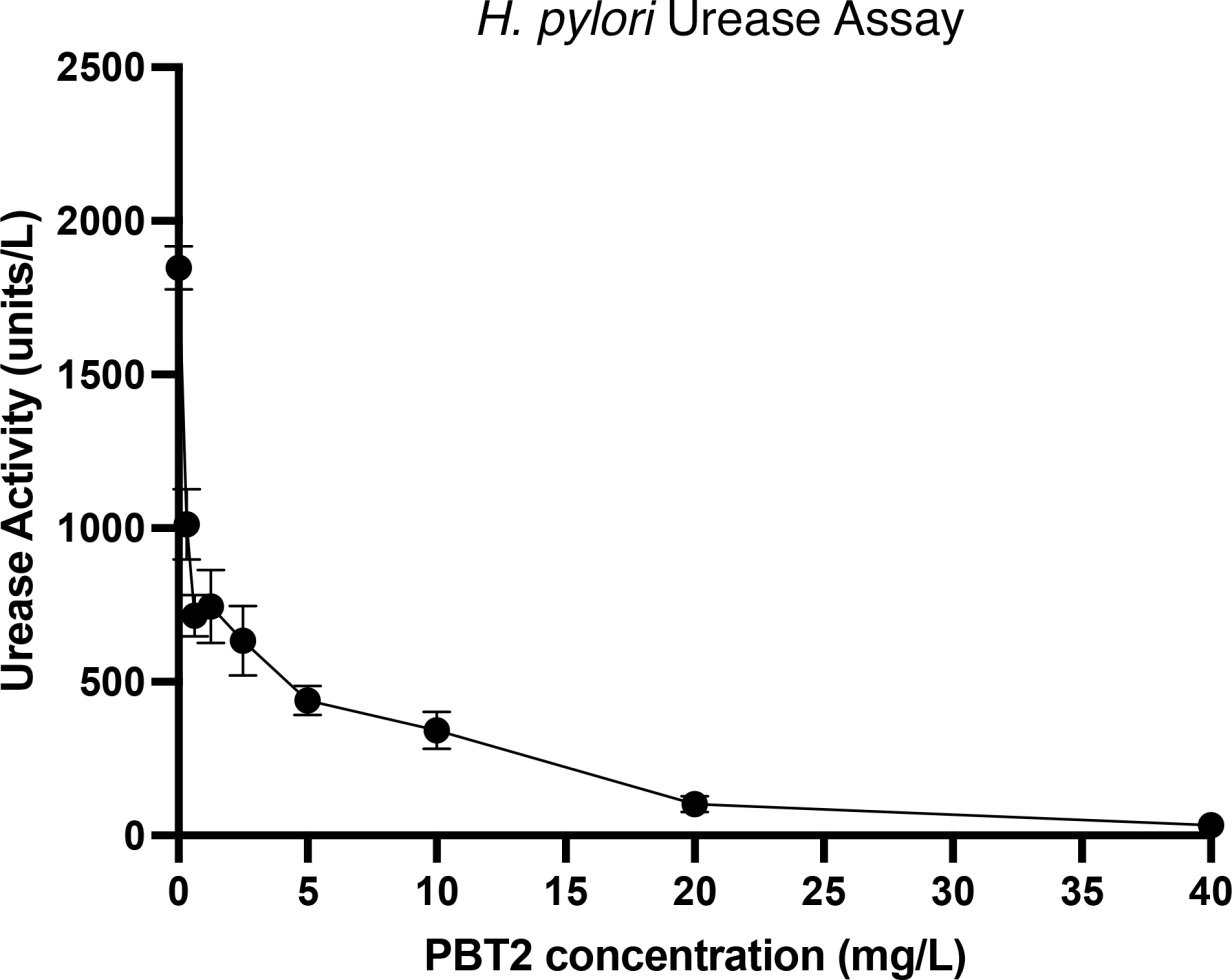
Dose-dependent inhibition of urease activity in *H. pylori* crude cell-free extracts by PBT2. Crude cell-free extracts of *H. pylori* were treated with PBT2 at concentrations ranging from 0.313 to 40 mg/L for 1 h at 37°C. Data represent the mean ± SD of triplicate measurements. The curve illustrates the dose-dependent decrease in urease activity upon PBT2 treatment.

To determine whether PBT2 can impact nickel-dependent urease enzymes, urease activity in *H. pylori* cell-free extracts was measured following exposure to increasing concentrations of PBT2 (Fig. 4). Urease activity was measured following removal of PBT2 using desalting columns, indicating that the observed inhibition was not due to direct interference with the assay reagents but reflects an effect on enzyme activity (see Materials and Methods). As shown in Fig. 4, urease activity in untreated controls ranged from ~1,800 to 2,000 units/L, consistent with previous reports describing the enzyme as highly abundant and essential for acid resistance and colonization (43, 44). PBT2 treatment resulted in a rapid reduction in urease activity in a concentration-dependent manner. The inhibition profile appeared in two phases, with a rapid initial decline in activity at low PBT2 concentrations followed by a more gradual reduction at higher concentrations, suggesting differential sensitivity of urease activity to perturbation of nickel homeostasis. At low concentrations (1 mg/L–2 mg/L), urease activity decreased by approximately 50%–60% relative to the untreated control. Further reductions were observed at intermediate concentrations (5 mg/L–10 mg/L), where activity declined to ~20%–30% of control levels. At higher concentrations (20 mg/L–40 mg/L), urease activity was almost completely abolished, reaching near-background levels (>90% inhibition).

These data provide direct functional evidence that PBT2 disrupts urease activity in *H. pylori*. Given that urease is a nickel-dependent enzyme requiring incorporation of Ni^2+^ into its active site for catalytic activity, the observed inhibition is consistent with perturbation of intracellular nickel homeostasis. This is consistent with previous studies showing that perturbation of nickel availability through chelation or altered intracellular trafficking impairs urease maturation and activity. The potent, dose-dependent inhibition observed here supports a mechanism in which PBT2, acting as a metal ionophore, alters nickel distribution and thereby compromises the activity of nickel-dependent enzymes.

## DISCUSSION

The increasing prevalence of antibiotic-resistant *H. pylori* has reduced the effectiveness of standard eradication regimens, highlighting the urgent need for alternative therapeutic strategies with novel mechanisms of action. In this study, we show that PBT2, an 8-hydroxyquinoline compound originally developed for neurodegenerative diseases, exhibits potent bactericidal activity against *H. pylori*, including multidrug-resistant clinical isolates. Together, our findings identify PBT2 as a promising repurposed candidate, combining rapid killing kinetics, a low propensity for resistance development, and efficacy in a murine infection model.

*In vitro*, PBT2 demonstrated rapid, concentration-dependent bactericidal activity, achieving complete killing of *H. pylori* within hours at concentrations close to the MIC, outperforming commonly used antibiotics under equivalent conditions. Importantly, prolonged exposure to subinhibitory concentrations of PBT2 over 30 days of sequential challenges did not result in the development of resistance, in contrast to the rapid resistance development observed with amoxicillin. Given the strong selective pressures within the gastric niche and the capacity of *H. pylori* to persist for decades in the human host, this apparent inability to readily adapt to PBT2 exposure may represent a clinically relevant advantage. However, this approach may not detect extremely rare spontaneous mutants arising at very low frequencies, and complementary methods, such as high-inoculum selection at supra-MICs, could be used in future studies to further assess the potential for single-step resistance emergence.

Proteomic analyses provide insight into the basis of this low resistance potential. SWATH-MS profiling revealed that PBT2 disrupts multiple essential cellular systems rather than acting on a single molecular target. Proteins involved in translation, ribosome structure, iron-sulfur cluster assembly, respiration, and metal acquisition were broadly suppressed following PBT2 exposure. These pathways are fundamental to bacterial viability and metabolic homeostasis, and their concurrent disruption is likely to limit the ability of *H. pylori* to mount effective compensatory responses. This multifactorial mode of action is consistent with previous studies of PBT2 in other bacterial pathogens and contrasts with conventional antibiotics that exert selective pressure on discrete targets.

The widespread dysregulation of metal-associated pathways observed here is consistent with the known ionophoric properties of PBT2. By redistributing transition metals such as zinc, copper, iron, and nickel, PBT2 is likely to interfere with metalloproteins and metal-dependent enzymatic processes required for respiration, redox balance, and acid adaptation. In *H. pylori*, nickel is particularly important, as it is an essential cofactor for urease, a key enzyme that enables survival in the acidic gastric environment by hydrolyzing urea to produce ammonia and carbon dioxide (39). Our ITC data demonstrate that PBT2 binds Ni^2+^ with high affinity, suggesting that intracellular nickel availability may be directly perturbed. Consistent with this, proteomic analysis revealed reduced abundance of urease-associated proteins, including the urease maturation factor HypB and accessory proteins UreF and UreG. Disruption of urease maturation would be expected to compromise acid resistance and impair gastric colonization. In parallel, suppression of iron-sulfur cluster proteins and respiratory chain components indicates broader metabolic stress, while reduced abundance of virulence-associated factors, including outer membrane adhesins, components of the Cag pathogenicity island, motility proteins, and the cell shape determinant MreB, suggests impaired colonization capacity and pathogenic potential.

Under sustained subinhibitory exposure, *H. pylori* exhibited limited adaptive responses, reflected by selective increases in proteins associated with quinone biosynthesis, lipoproteins, and urease accessory functions. However, ribosomal proteins remained suppressed, indicating that full restoration of translational capacity does not occur. This constrained adaptive response may help explain the lack of resistance emergence during prolonged PBT2 exposure, as key recovery pathways remain functionally restricted.

The biological relevance of these findings is supported by the *in vivo* efficacy of PBT2 in a murine gastric infection model. Short-course oral administration significantly reduced gastric *H. pylori* burden, with most treated animals becoming culture-negative, while untreated and vehicle-treated controls remained colonized. Although murine models do not fully recapitulate the complexity of human infection, these data demonstrate that PBT2 retains antimicrobial activity within the gastric environment and can substantially reduce bacterial colonization *in vivo*.

While our study provides evidence of *in vivo* activity, several limitations should be considered. The BALB/c model and the *H. pylori* strain ATCC 700392 (26695), which have been used in previous studies (36–38), are known to support lower colonization levels compared to C57BL/6 mice or mouse-adapted strains such as SS1 (34). These host-pathogen factors likely contribute to the relatively low bacterial loads observed in this study. In addition, the short treatment duration (3 days) may limit conclusions regarding sustained clearance. Future studies using mouse-adapted strains, higher-colonization models, and extended treatment regimens will help to further define the *in vivo* efficacy and treatment parameters of PBT2. In addition, bacterial isolates recovered from the murine infection model were not retained for post-treatment susceptibility testing, preventing assessment of reduced PBT2 susceptibility following *in vivo* exposure. Future dose-ranging studies will incorporate isolate recovery to further evaluate resistance emergence under treatment conditions.

From a translational perspective, PBT2 is particularly attractive as a repurposing candidate given its prior clinical evaluation. In human trials for neurodegenerative indications, once-daily oral doses of up to 250 mg administered for up to six months were generally well tolerated, with no serious adverse events clearly attributable to treatment (17). The murine dosing regimen used in this study (60 mg/kg/day) corresponds to a human equivalent dose of approximately 340 mg/day for a 70 kg adult, which is of the same order of magnitude as doses assessed clinically. At this dose, systemic plasma exposure to PBT2 is relatively low (AUC ~1,660 ng·h/mL) (45) compared with the *H. pylori* MIC of approximately 1.25 mg/L. However, plasma concentrations may not accurately reflect drug levels achieved at the site of infection in the gastric mucosa, particularly for an orally administered compound. It is therefore plausible that higher local concentrations are achieved in the stomach, contributing to the *in vivo* efficacy observed. Nonetheless, direct measurements of gastric exposure are lacking, and further pharmacokinetic-pharmacodynamic studies will be required to define gastric drug levels and optimize delivery strategies.

In summary, this study demonstrates that PBT2 exerts potent, rapid, and sustained antibacterial activity against *H. pylori* through a multifaceted, metal-dependent mechanism that disrupts multiple essential cellular processes. The combination of rapid bactericidal activity, a low propensity for resistance development, *in vivo* efficacy, and established clinical safety supports further evaluation of PBT2 as a repurposed therapeutic for multidrug-resistant *H. pylori* infections. More broadly, these findings reinforce the potential of drug repurposing strategies to strengthen the antimicrobial pipeline by leveraging clinically advanced compounds with unconventional mechanisms of action.

## MATERIALS AND METHODS

### Bacterial strains and culture conditions

*H. pylori* strains, including reference strains ATCC 43526, ATCC 700392 (32), J99 (33), and SS1 (34), and clinical isolates (CH428 and CH426) (35), were used in this study. All strains were routinely cultured on Mueller-Hinton (Sigma-Aldrich) agar supplemented with 10% (vol/vol) of defibrinated horse blood. For broth-based assays, bacteria were cultured in BHI broth supplemented with 10% vol/vol of fetal bovine serum. Cultures were incubated under microaerophilic conditions (10% CO₂, 5% O₂, 85% N₂) (30) by using a gas-generating sachet in a sealed jar (Oxoid) at 37°C for 72 h.

### Minimal inhibitory concentrations

The MICs were determined using broth microdilution. Bacterial suspensions were adjusted to 1 × 10^6^ CFU/mL and incubated in a 96-well plate with twofold serial dilutions of PBT2. The cell density of *H. pylori* at an OD₆₀₀ of 1.0 was determined by viable counting on blood agar plates and was approximately 2 × 10⁸ CFU/mL. Negative wells (medium only) and positive wells (no drug, bacteria only) were used as controls. Tetracycline and amoxicillin were included as positive drug controls in all assays. MICs were also determined using the agar diffusion method, as described in CLSI guidelines (31). Briefly, PBT2 was twofold serially diluted in molten MH agar supplemented with 5% (vol/vol) defibrinated sheep blood. *H. pylori* suspension was adjusted to 1 × 10⁷ CFU/mL, and a 2 μL aliquot of each bacterial suspension was inoculated onto the surface of each drug-containing agar plate. A control plate without PBT2 was also included. All plates were incubated at 37°C for 72 h. MIC values were determined as the lowest concentration of PBT2 that completely inhibited the visible growth of bacteria after incubation. Each experiment was performed at least twice in biological triplicate to confirm the results.

### Time-kill kinetics assay

Time-kill assays were performed as described in CLSI guidelines (46). *H. pylori* cultures were exposed to PBT2 at 0.5×, 1×, and 2× MICs, and bacterial viability was assessed at 0, 4, 8, 10, 12, 24, 48, and 72 h by removing 10 μL aliquots and serially diluting in phosphate-buffered saline (PBS) and spot plating onto blood agar. Amoxicillin and tetracycline were included as positive controls. The negative control group with no drug treatment was also included. Plates were incubated under microaerobic conditions at 37°C for 72 h. CFU were counted and presented as log_10_ CFU/mL versus incubation time. The experiments were performed at least twice in biological triplicate.

### Drug-resistant development assay

Serial passage experiments were conducted to evaluate the potential for resistance development to PBT2 as previously described (29). Briefly, *H. pylori* ATCC 43526, CH428, and J99 were used in this assay. A twofold dilution of PBT2 in a 96-well plate was first set up, and the bacteria inoculum concentration was adjusted to 1 × 10^6^ CFU/mL. The positive drug control group was amoxicillin for ATCC 43526. A negative control with no drug treatment was also included. The MIC was checked every 3 days for a total of 30 days. Cells from the highest PBT2 or amoxicillin concentrations that showed growth after 72 h of incubation were sub-cultured into a new microtiter plate with twofold dilutions of PBT2 or amoxicillin. The drug concentration was adjusted with changes in the MIC. Serial passaging was performed in biological triplicate for each strain and treatment condition.

### *In vivo* animal study

Seven-week-old male BALB/c mice were obtained from the National Laboratory Animal Center of Taiwan. *H. pylori* strain ATCC 700392 (26695) was used for the mouse infection experiments as previously described (34, 36–38). Mice were randomly assigned to four groups: (i) *H. pylori* infection only (*n* = 6), (ii) *H. pylori* + SSV treatment (*n* = 6), and (iii) *H. pylori* + PBT2 treatment (*n* = 7). For experimental infection, mice were inoculated with *H. pylori* (100 μL of 1 × 10⁹ CFU/mL) via intragastric gavage once daily for 7 consecutive days (days 1–7). Following the final inoculation, SSV or PBT2 was administered twice daily for 3 consecutive days (days 8–10). On day 12 (2 days post-treatment), mice were euthanized for tissue collection. The solvent, SSV, consisting of 0.9% wt/vol NaCl, 0.5% wt/vol sodium carboxymethylcellulose, 0.5% vol/vol benzyl alcohol, and 0.4% vol/vol Tween-80, was used for *in vivo* administration of PBT2. The stomach tissues were homogenized, and serial dilutions were plated on selective agar and incubated under microaerophilic conditions for 3 days to quantify *H. pylori* colonization. Bacterial load was expressed as colony-forming units per milligram of tissue (CFU/mg). One-way analysis of variance and Dunnett’s multiple comparison tests were performed using GraphPad Prism (version 10.2, GraphPad Software, USA). The data were presented as mean ± SD. Statistical significance was defined as *P < 0.05, **P < 0.01, ***P < 0.001, and ****P < 0.0001.

### Peptide preparation for SWATH proteomics analysis

ATCC 43526 *H. pylori* were grown in six-well plates with an initial inoculum of ~5 × 10^5^ to 1 × 10^6^ CFU/mL and harvested at time points of 0 h, 8 h, 10 h, and 12 h after exposure to PBT2 at 0.5×, 1×, and 2× MICs.

Protein samples were processed using the S-Trap micro spin column digestion as per instructions by the manufacturer (ProtiFi, LLC). Briefly, pelleted samples were resuspended in 5% SDS, 50 mM TEAB, pH 8.5, and sonicated for 5 min. Samples were subsequently clarified by centrifugation at 13,000 × *g* for 8 min to remove insoluble debris. DTT was added to a final concentration of 5 mM and incubated at 56°C for 30 min. Iodoacetamide was added to a final concentration of 25 mM, and the mixture was incubated for 30 min at room temperature in a dark environment. Proteins were precipitated by acidification with a final phosphoric acid concentration of 2.5%.

Denatured proteins were trapped using S-Trap micro spin columns (ProtiFi, LLC). Binding was achieved by adding binding/wash buffer (90% methanol, 100 mM TEAB, pH 7.55) to the acidified protein solution. Samples were then transferred onto the S-Trap column and centrifuged at 4,000 × *g* for 30 s. Washes were performed three times with binding/wash buffer, each followed by centrifugation at 4,000 × *g* for 30 s.

Samples were then treated with digestion buffer containing 50 mM TEAB, pH 8.5, and trypsin and incubated at 37°C overnight. Peptides were sequentially eluted from the S-Trap columns by centrifugation (4,000 × *g*, 1 min) using elution buffer containing 50 mM TEAB, pH 8.5, 0.2% formic acid, and 50% acetonitrile. The eluted peptides were dried using SpeedVac and resuspended in 0.1% formic acid for LC-MS/MS analysis.

### SWATH-MS

SWATH-MS was performed as described previously (47). Desalted peptides were analyzed by LC-ESI-MS/MS using an M-Class UPLC system (Waters) coupled to a ZenoTof 7600 instrument (Sciex, Massachusetts, USA). Peptides were separated using a Waters NanoEase HSS T3 column (100 Å, 1.8 µm, 300 µm × 150 mm) at a flow rate of 5 μL/min at 40°C. Chromatography was performed with buffer A (0.1% formic acid in water) and buffer B (0.1% formic acid in acetonitrile) under the following gradient: 5% B for 0–0.6 min, 5%–35% B from 0.6 to 22 min, 35%–90% B from 22 to 23 min, held at 90% B for 3 min, and re-equilibrated at 5% B for 4 min, for a total run time of 30 min per sample. Proteins were detected and quantified using data-independent acquisition (DIA) SWATH-MS (48). An MS TOF scan was acquired over 400–1,500 m/z for 0.1 s, and variable windows spanning 399.5 m/z–750.5 m/z for 0.013 s were fragmented. MS/MS spectra were collected across 140–1,750 m/z, with an intensity threshold of 100,000 cps. Dynamic collision energy was applied. Gas and voltage were adjusted as required. Peptides were identified using DIA-NN (version 1.8, Sciex) searching against the UniProt reference proteome of *H. pylori* (UP000000429, downloaded from UniProt KB on 20 May 2024). Peptide and protein quantification was performed using PeakView (version 2.2, Sciex), and protein abundances were recalculated and filtered to a 1% false discovery rate threshold.

UniProt protein IDs were mapped to protein names using the UniProt Retrieve/ID mapping tool (https://www.uniprot.org/). Differential analyses were performed using MSstats in R (49), using an adjusted significance threshold of *P*  =  10^−5^. Clustered heatmap and GO term enrichment analysis were performed using R and R Studio (v.4.5, The R Foundation).

### ITC

ITC was performed as described previously (50). Zinc sulfate heptahydrate, copper(II) sulfate pentahydrate, nickel(II) sulfate hexahydrate, and magnesium sulfate were purchased from Sigma-Aldrich as ACS reagent grade. ITC measurements were performed using a Nano ITC (TA Instruments) in triplicate at 37°C in a buffer containing 10 mM HEPES, 150 mM NaCl, and 4% DMSO at pH 7.4. For the zinc experiment, 170 μL of 0.25 mM PBT2 was loaded into the cell. One microliter × 40 injections of 0.75 mM zinc solutions were titrated. Copper, nickel, and magnesium experiments: 170 μL of 0.25 mM PBT2 was loaded into the cell. A total of 2.5 μL × 15 injections of 2 mM metal solutions were titrated. The buffer pH for the copper experiment was adjusted to 6.4 to prevent precipitation. The interval between each injection was set to 200 s. Blank control groups (metal titrate into buffer only) were also included to subtract heat dilution effects from each ITC experiment. All the solutions were degassed before the titrations were performed. Data were analyzed using the built-in TA NanoAnalyse software and fitted to the independent binding model. Data were presented as the mean ± SD from three independent experiments.

### ICP-MS

ICP-MS experiment was performed as described previously (24). ATCC 43526 OD_600_ of 0.5 cells were treated with and without PBT2 (1.25 mg/L) for 1 h at 37°C under a microaerophilic environment. Cells were harvested at 3,200 × *g* for 10 min at 4°C. The pellets were washed three times with PBS containing 5 mM EDTA, followed by three additional washes with PBS without EDTA. Blank samples were also included. Aliquots were saved for subsequent BCA measurement. Samples were then digested with 70% nitric acid at 95°C for 1 h, followed by incubation at 85°C for 24 h. Samples were diluted with HPLC-grade water to a final concentration of 2% nitric acid. Samples were analyzed on an Agilent 8900 Triple Quadrupole ICP-MS operating in helium collision mode. Three biological replicates were analyzed. Metal levels were converted using the corresponding atomic masses and normalized to total protein content. Data are presented as mean ± SD of triplicates. Statistical significance was calculated using an unpaired two-tailed *t*-test, using GraphPad Prism (version 10.2, GraphPad Software, USA). Statistical significance was defined as *P < 0.05, **P < 0.01, ***P < 0.001, and ****P < 0.0001.

### Urease activity assay

*H. pylori* cells were harvested after 72 h of growth on BHI blood agar plates and resuspended in ice-cold sodium phosphate buffer (20 mM, pH 7.0). The cell suspension was adjusted to an OD_600_ of 0.5. Cell-free extracts were prepared using 0.1 mm glass beads and a bead beater (Tissuelyser LT) operating at 50 oscillation cycles for 3 min over 4 cycles at 4°C. After lysis, the cell-free extracts were carefully transferred to new tubes, avoiding the transfer of glass beads. Crude urease lysates were then centrifuged at 18,000 × *g* for 10 min at 4°C to remove debris and any remaining beads. The supernatant was collected and treated with PBT2 in a twofold serial dilution ranging from 40 mg/L to 0.313 mg/L, followed by incubation at 37°C for 1 h. A control group without PBT2 was also included. After incubation, desalting columns (Thermo Fisher, Zeba 7K MWCO) were used to remove PBT2. Samples were then diluted 1:40 in assay buffer and prepared in triplicate. Urease activity was measured according to the manufacturer’s instructions using a commercial kit (Thermo Fisher, MAK120), based on the Berthelot method. The urease activity (units/L) is calculated as:


$$\text{Urease activity}\left(\frac{\text{units}}{\text{L}}\right)=\frac{\left(A_{670}sample-A_{670}blank\right)\times n}{\text{Slope}\times t}$$

where *n* is the dilution factor (40), and *t* is the urea incubation time (10 min).

## SUPPLEMENTAL MATERIAL

Figure S1 mbio.01229-26-s0001.tif ITC analysis of PBT2 and metal binding at 37°C.

Supplemental material mbio.01229-26-s0002.docx Figure S1 legend; Table S1.

ASM does not own the copyrights to Supplemental Material that may be linked to, or accessed through, an article. The authors have granted ASM a non-exclusive, world-wide license to publish the Supplemental Material files. Please contact the corresponding author directly for reuse.
