# Supplementary figures and images for: Ionophore PBT2 as a novel approach to combat antibiotic-resistant Helicobacter pylori
Source: mBio. 2026 Jun 15;17(7):e01229-26. doi: 10.1128/mbio.01229-26 (PMC13343840; doi:10.1128/mbio.01229-26)

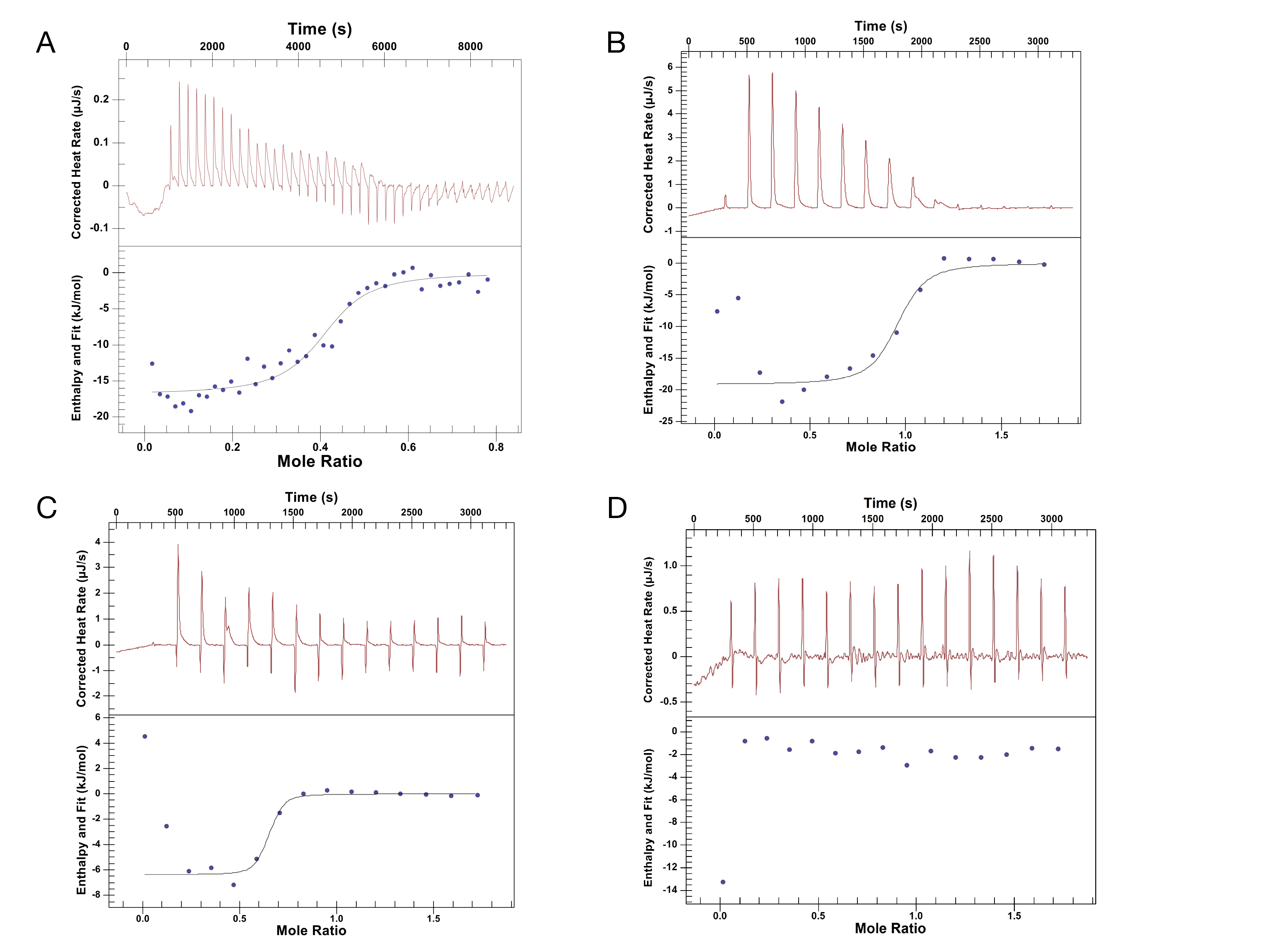

Supplement: Figure S1 — ITC analysis of PBT2 and metal binding at 37°C. [file mbio.01229-26-s0001.tif]
