# Supplementary material for: Ionophore PBT2 as a novel approach to combat antibiotic-resistant Helicobacter pylori
Source: mBio. 2026 Jun 15;17(7):e01229-26. doi: 10.1128/mbio.01229-26 (PMC13343840; doi:10.1128/mbio.01229-26)
Supplement: Supplemental material — Figure S1 legend; Table S1. [file mbio.01229-26-s0002.docx]

**Supplementary Materials:**

**Figure legend**

Figure S1. ITC analysis of PBT2 and metal binding at 37 °C. (A) 0.75 mM Zn into 0.25 mM PBT2 in 10 mM HEPES and 150 mM NaCl at pH 7.4. (B) 2 mM Cu into 0.25 mM PBT2 in 10 mM HEPES and 150 mM NaCl at pH 6.4. (C) 2 mM Ni into 0.25mM PBT2 in 10 mM HEPES and 150 mM NaCl at pH 7.4. (D) 2 mM Mg into 0.25 mM PBT2 in 10 mM HEPES and 150 mM NaCl at pH 7.4. The top panel shows the raw ITC data collected from the nano ITC. The curve represents the best fit to an independent single site model.

**Table S1.** Differentially regulated proteins (*adj.p*<0.05, FC>2) in ATCC 43526 *H. pylori* cells treated with PBT2 at T8, T10 and T12 under MIC concentration (1.25 mg/L) or sub-inhibitory MIC concentration (0.625 mg/L).

| Protein | Annotation | log2FC | Fold Change | adj. pvalue |
| --- | --- | --- | --- | --- |
| Increased abundance in PBT2 treated cells at T8 MIC concentration (1.25 mg/L) | | | | |
| O24965 | AMIN domain-containing protein | 2.04356819 | 4.12263916 | 0.03149616 |
| O25280 | Sialidase | 1.85278618 | 3.61197067 | 0.0179044 |
| O25729 | 2-dehydro-3-deoxy-phosphogluconate aldolase (EC 4.1.2.14) | 1.32832668 | 2.51111252 | 3.60E-06 |
| O26102 | Pyridoxine 5'-phosphate synthase (PNP synthase) (EC 2.6.99.2) | 1.20525743 | 2.3057841 | 0.01496605 |
| O25927 | Acyl-[acyl-carrier-protein] --UDP-N-acetylglucosamine O-acyltransferase (UDP-N-acetylglucosamine acyltransferase) (EC 2.3.1.129) | 1.03172525 | 2.04446767 | 0.00478041 |
| O25863 | NADH-quinone oxidoreductase subunit N (EC 7.1.1.-) (NADH dehydrogenase I subunit N) (NDH-1 subunit N) | 1.00191709 | 2.00265942 | 0.0003607 |
| Decreased abundance in PBT2 treated cells at T8 MIC concentration (1.25 mg/L) | | | | |
| P56021 | Small ribosomal subunit protein uS14 (30S ribosomal protein S14 type Z) | -1.8564798 | 3.62123008 | 0.00044343 |
| P56020 | Small ribosomal subunit protein uS13 (30S ribosomal protein S13) | -1.747047 | 3.35670783 | 3.55E-07 |
| P0A0X4 | Small ribosomal subunit protein uS12 (30S ribosomal protein S12) | -1.7423461 | 3.34578819 | 0 |
| O24950 | Conserved hypothetical iron-sulfur protein | -1.7274044 | 3.3113153 | 8.89E-08 |
| O25009 | Nitrogen fixation protein NifU | -1.7101965 | 3.27205396 | 0 |
| O25088 | Sec-independent protein translocase protein TatA | -1.6465126 | 3.1307594 | 1.58E-14 |
| O25362 | Soluble lytic murein transglycosylase (Slt) | -1.5622433 | 2.95312689 | 0.00015588 |
| P56040 | Large ribosomal subunit protein uL15 (50S ribosomal protein L15) | -1.5599737 | 2.9484846 | 1.74E-13 |
| P56049 | Large ribosomal subunit protein uL24 (50S ribosomal protein L24) | -1.5282063 | 2.88427021 | 1.07E-06 |
| P66142 | Large ribosomal subunit protein bL28 (50S ribosomal protein L28) | -1.5174806 | 2.86290654 | 0 |
| P55973 | Translation initiation factor IF-3 | -1.4391908 | 2.71168733 | 6.40E-05 |
| P56045 | Large ribosomal subunit protein bL20 (50S ribosomal protein L20) | -1.4192436 | 2.67445253 | 0 |
| P56042 | Large ribosomal subunit protein bL17 (50S ribosomal protein L17) | -1.3862886 | 2.61405331 | 3.90E-13 |
| P64275 | Transcription elongation factor GreA (Transcript cleavage factor GreA) | -1.3852013 | 2.61208398 | 0.00380503 |
| O25008 | Cysteine desulfurase IscS (EC 2.8.1.7) | -1.3821964 | 2.60664922 | 0 |
| P56026 | Small ribosomal subunit protein uS19 (30S ribosomal protein S19) | -1.3599088 | 2.56668953 | 1.45E-11 |
| O25858 | NADH-quinone oxidoreductase subunit I (EC 7.1.1.-) (NADH dehydrogenase I subunit I) (NDH-1 subunit I) | -1.321839 | 2.49984556 | 0.00217556 |
| P56047 | Large ribosomal subunit protein uL22 (50S ribosomal protein L22) | -1.3177549 | 2.49277884 | 0 |
| P56103 | Guanylate kinase (EC 2.7.4.8) (GMP kinase) | -1.2925451 | 2.44959812 | 0.01842644 |
| O26052 | Lipoprotein | -1.2638852 | 2.40141574 | 1.24E-07 |
| P56466 | Transcription termination factor Rho (EC 3.6.4.-) (ATP-dependent helicase Rho) | -1.2612519 | 2.39703652 | 0 |
| O06914 | Fumarate reductase iron-sulfur subunit (EC 1.3.5.1) (Quinol-fumarate reductase iron-sulfur subunit) (QFR iron-sulfur subunit) | -1.25242 | 2.38240719 | 1.55E-06 |
| P66449 | Small ribosomal subunit protein uS17 (30S ribosomal protein S17) | -1.2472005 | 2.37380353 | 0 |
| P56036 | Large ribosomal subunit protein uL10 (50S ribosomal protein L10) | -1.2403279 | 2.3625223 | 0 |
| P56046 | Large ribosomal subunit protein bL21 (50S ribosomal protein L21) | -1.2366234 | 2.35646355 | 5.54E-15 |
| O24959 | DUF4006 family protein | -1.235091 | 2.35396195 | 0.00916902 |
| P56034 | Large ribosomal subunit protein uL6 (50S ribosomal protein L6) | -1.2329976 | 2.35054882 | 0 |
| O24951 | Conserved hypothetical secreted protein | -1.2298522 | 2.34542961 | 0.00065314 |
| P66621 | Small ribosomal subunit protein uS8 (30S ribosomal protein S8) | -1.1997261 | 2.2969606 | 0 |
| O25395 | Iron (III) dicitrate transport protein (FecA) | -1.1935556 | 2.2871573 | 0.01276476 |
| P56032 | Large ribosomal subunit protein uL4 (50S ribosomal protein L4) | -1.1694618 | 2.24927776 | 3.65E-10 |
| P56029 | Large ribosomal subunit protein uL1 (50S ribosomal protein L1) | -1.1653277 | 2.24284151 | 0 |
| O25761 | Protein CR006 P-loop domain-containing protein | -1.1590214 | 2.23305904 | 7.07E-09 |
| O24911 | Methyl-accepting chemotaxis transducer (TlpC) | -1.1550966 | 2.2269923 | 0.00015588 |
| P66119 | Large ribosomal subunit protein uL23 (50S ribosomal protein L23) | -1.1537824 | 2.22496457 | 0 |
| O25503 | Polyamine aminopropyltransferase (Putrescine aminopropyltransferase) (PAPT) (Spermidine synthase) (SPDS) (SPDSY) (EC 2.5.1.16) | -1.1521074 | 2.22238293 | 1.14E-12 |
| P52093 | Bacterial non-heme ferritin (EC 1.16.3.2) | -1.1000092 | 2.14356059 | 0 |
| P56013 | Small ribosomal subunit protein bS6 (30S ribosomal protein S6) | -1.0978401 | 2.14034022 | 2.63E-11 |
| O25286 | 3-oxoacyl-[acyl-carrier-protein] reductase (EC 1.1.1.100) | -1.0850899 | 2.12150772 | 0 |
| O26032 | Copper resistance protein CopD | -1.064464 | 2.0913928 | 0.0061363 |
| P94845 | Glutamine synthetase (GS) (EC 6.3.1.2) (Glutamate--ammonia ligase) (Glutamine synthetase I beta) (GSI beta) | -1.0636943 | 2.09027716 | 4.64E-06 |
| P56044 | Large ribosomal subunit protein bL19 (50S ribosomal protein L19) | -1.0523726 | 2.07393778 | 0 |
| P56033 | Large ribosomal subunit protein uL5 (50S ribosomal protein L5) | -1.0394544 | 2.05545016 | 1.54E-11 |
| P56039 | Large ribosomal subunit protein uL14 (50S ribosomal protein L14) | -1.039435 | 2.05542247 | 3.06E-09 |
| P56010 | Small ribosomal subunit protein uS3 (30S ribosomal protein S3) | -1.0270858 | 2.03790358 | 0 |
| P66328 | Small ribosomal subunit protein uS10 (30S ribosomal protein S10) | -1.020916 | 2.02920693 | 1.92E-11 |
| O24864 | Chemotaxis protein CheV1 | -1.0087493 | 2.01216599 | 0.00024082 |
| P66637 | Small ribosomal subunit protein uS9 (30S ribosomal protein S9) | -1.0055184 | 2.00766484 | 7.75E-08 |
| Increased abundance in PBT2 treated cells at T10 Sub-inhibitory MIC concentration (0.625 mg/L) | | | | |
| O25973 | Histidine and glutamine-rich protein | 2.35884237 | 5.12958592 | 4.14E-07 |
| O25280 | Sialidase | 1.17117152 | 2.25194489 | 0.01106853 |
| Decreased abundance in PBT2 treated cells at T10 Sub-inhibitory MIC concentration (0.625 mg/L) | | | | |
| P66637 | Small ribosomal subunit protein uS9 (30S ribosomal protein S9) | -2.2927574 | 4.8999173 | 0 |
| P56040 | Large ribosomal subunit protein uL15 (50S ribosomal protein L15) | -2.276531 | 4.84511531 | 0 |
| O25761 | Protein CR006 P-loop domain-containing protein | -2.2444977 | 4.73872093 | 9.94E-07 |
| P56071 | Threonine--tRNA ligase (EC 6.1.1.3) (Threonyl-tRNA synthetase) (ThrRS) | -2.126115 | 4.36540347 | 5.83E-06 |
| P56046 | Large ribosomal subunit protein bL21 (50S ribosomal protein L21) | -2.1192847 | 4.34478474 | 0 |
| O25088 | Sec-independent protein translocase protein TatA | -2.107725 | 4.31011093 | 0 |
| O25858 | NADH-quinone oxidoreductase subunit I (EC 7.1.1.-) (NADH dehydrogenase I subunit I) (NDH-1 subunit I) | -2.0054355 | 4.01509883 | 2.35E-07 |
| P56049 | Large ribosomal subunit protein uL24 (50S ribosomal protein L24) | -1.9931321 | 3.98100339 | 4.58E-09 |
| O25142 | Alpha-(1,3)-fucosyltransferase (EC 2.4.1.152) (4-galactosyl-N-acetylglucosaminide 3-alpha-L-fucosyltransferase) | -1.9396465 | 3.83611641 | 0.00097753 |
| P56042 | Large ribosomal subunit protein bL17 (50S ribosomal protein L17) | -1.9163166 | 3.77458125 | 0 |
| P56030 | Large ribosomal subunit protein uL2 (50S ribosomal protein L2) | -1.8684585 | 3.65142222 | 0 |
| P52093 | Bacterial non-heme ferritin (EC 1.16.3.2) | -1.8624609 | 3.63627397 | 0 |
| O24884 | mannose-1-phosphate guanylyltransferase (EC 2.7.7.13) | -1.8472126 | 3.59804343 | 4.38E-08 |
| P56466 | Transcription termination factor Rho (EC 3.6.4.-) (ATP-dependent helicase Rho) | -1.8321652 | 3.56071064 | 0 |
| P66142 | Large ribosomal subunit protein bL28 (50S ribosomal protein L28) | -1.819405 | 3.5293561 | 0 |
| O25769 | Flagellar assembly factor FliW 1 | -1.8192914 | 3.5290782 | 4.28E-08 |
| P55973 | Translation initiation factor IF-3 | -1.8163682 | 3.5219348 | 8.60E-08 |
| O25531 | D-glycero-beta-D-manno-heptose-1,7-bisphosphate 7-phosphatase (EC 3.1.3.82) (D,D-heptose 1,7-bisphosphate phosphatase) (HBP phosphatase) | -1.7936358 | 3.46687495 | 0.00149082 |
| P56026 | Small ribosomal subunit protein uS19 (30S ribosomal protein S19) | -1.7850933 | 3.44640751 | 4.11E-13 |
| P56033 | Large ribosomal subunit protein uL5 (50S ribosomal protein L5) | -1.7816073 | 3.43808997 | 0 |
| O25008 | Cysteine desulfurase IscS (EC 2.8.1.7) | -1.7803596 | 3.43511786 | 0 |
| P56078 | Large ribosomal subunit protein bL25 (50S ribosomal protein L25) (General stress protein CTC) | -1.7772489 | 3.42771913 | 0 |
| P0A0X4 | Small ribosomal subunit protein uS12 (30S ribosomal protein S12) | -1.7745647 | 3.42134763 | 0 |
| P55977 | Transcription termination/antitermination protein NusA | -1.7422453 | 3.34555439 | 6.45E-06 |
| O25873 | Conserved hypothetical secreted protein | -1.7231877 | 3.30165116 | 0 |
| P56034 | Large ribosomal subunit protein uL6 (50S ribosomal protein L6) | -1.7139925 | 3.28067459 | 0 |
| P56045 | Large ribosomal subunit protein bL20 (50S ribosomal protein L20) | -1.7079258 | 3.26690794 | 0 |
| P56041 | Large ribosomal subunit protein uL16 (50S ribosomal protein L16) | -1.7077704 | 3.26655606 | 0 |
| P56029 | Large ribosomal subunit protein uL1 (50S ribosomal protein L1) | -1.701472 | 3.25232628 | 0 |
| P56020 | Small ribosomal subunit protein uS13 (30S ribosomal protein S13) | -1.6981273 | 3.24479492 | 2.12E-14 |
| O26042 | Iron-regulated outer membrane protein (FrpB) | -1.6847643 | 3.21487869 | 0 |
| P56031 | Large ribosomal subunit protein uL3 (50S ribosomal protein L3) | -1.6780698 | 3.19999533 | 1.23E-14 |
| O25009 | Nitrogen fixation protein NifU | -1.668554 | 3.1789581 | 0 |
| P56036 | Large ribosomal subunit protein uL10 (50S ribosomal protein L10) | -1.6548331 | 3.1488676 | 0 |
| O25536 | Deoxyuridine 5'-triphosphate nucleotidohydrolase (dUTPase) (EC 3.6.1.23) (dUTP pyrophosphatase) | -1.6491148 | 3.13641138 | 3.83E-06 |
| P66621 | Small ribosomal subunit protein uS8 (30S ribosomal protein S8) | -1.6382173 | 3.11280953 | 0 |
| P56047 | Large ribosomal subunit protein uL22 (50S ribosomal protein L22) | -1.6378878 | 3.11209867 | 0 |
| P56038 | Large ribosomal subunit protein uL13 (50S ribosomal protein L13) | -1.6008162 | 3.03314864 | 0 |
| O26082 | Iron(III) ABC transporter, periplasmic iron-binding protein (CeuE) | -1.5942339 | 3.01934141 | 9.12E-06 |
| P56010 | Small ribosomal subunit protein uS3 (30S ribosomal protein S3) | -1.5836714 | 2.99731643 | 0 |
| P94845 | Glutamine synthetase (GS) (EC 6.3.1.2) (Glutamate--ammonia ligase) (Glutamine synthetase I beta) (GSI beta) | -1.5823572 | 2.99458732 | 1.42E-12 |
| O26031 | Outer membrane protein (Omp32) | -1.5793994 | 2.98845413 | 1.10E-07 |
| O25286 | 3-oxoacyl-[acyl-carrier-protein] reductase (EC 1.1.1.100) | -1.5790062 | 2.98763975 | 0 |
| P56039 | Large ribosomal subunit protein uL14 (50S ribosomal protein L14) | -1.5664014 | 2.9616505 | 0 |
| P56044 | Large ribosomal subunit protein bL19 (50S ribosomal protein L19) | -1.5655963 | 2.9599982 | 0 |
| P56022 | Small ribosomal subunit protein uS15 (30S ribosomal protein S15) | -1.5540553 | 2.93641383 | 2.74E-14 |
| P55834 | Large ribosomal subunit protein bL12 (50S ribosomal protein L7/L12) | -1.5402111 | 2.90837057 | 0 |
| P66119 | Large ribosomal subunit protein uL23 (50S ribosomal protein L23) | -1.5381082 | 2.90413436 | 0 |
| P66609 | Small ribosomal subunit protein uS7 (30S ribosomal protein S7) | -1.5360705 | 2.90003538 | 0 |
| P66052 | Large ribosomal subunit protein uL11 (50S ribosomal protein L11) | -1.5216163 | 2.87112531 | 0 |
| P55976 | Transcription termination/antitermination protein NusG | -1.500538 | 2.82948208 | 0 |
| P56124 | Proline--tRNA ligase (EC 6.1.1.15) (Prolyl-tRNA synthetase) (ProRS) | -1.4973348 | 2.82320678 | 3.23E-10 |
| O25997 | cytochrome-c peroxidase (EC 1.11.1.5) | -1.4848975 | 2.79897286 | 0.01203594 |
| O24886 | GDP-L-fucose synthase (EC 1.1.1.271) (GDP-4-keto-6-deoxy-D-mannose-3,5-epimerase-4-reductase) | -1.4690179 | 2.76833378 | 2.64E-05 |
| P66449 | Small ribosomal subunit protein uS17 (30S ribosomal protein S17) | -1.4678722 | 2.76613621 | 0 |
| P56009 | Small ribosomal subunit protein uS2 (30S ribosomal protein S2) | -1.4650814 | 2.76079047 | 0 |
| O24951 | Conserved hypothetical secreted protein | -1.4644551 | 2.75959222 | 1.70E-12 |
| O26032 | Copper resistance protein CopD | -1.4618622 | 2.75463697 | 1.63E-07 |
| Q09065 | Urease accessory protein UreF | -1.4540086 | 2.73968229 | 9.58E-08 |
| O25262 | Cag pathogenicity island protein (Cag7) | -1.4524345 | 2.7366947 | 3.66E-06 |
| P56011 | Small ribosomal subunit protein uS4 (30S ribosomal protein S4) | -1.4480983 | 2.72848157 | 0 |
| P56050 | Large ribosomal subunit protein bL27 (50S ribosomal protein L27) | -1.4361917 | 2.70605601 | 6.78E-14 |
| P66328 | Small ribosomal subunit protein uS10 (30S ribosomal protein S10) | -1.4253356 | 2.6857697 | 0 |
| P66572 | Small ribosomal subunit protein uS5 (30S ribosomal protein S5) | -1.4172591 | 2.67077622 | 0 |
| O25501 | Ss-DNA binding protein 12RNP2 | -1.4119384 | 2.66094446 | 1.87E-10 |
| O24944 | Sodium:calcium antiporter | -1.4044413 | 2.64715248 | 0 |
| O24968 | Putative beta-lactamase HcpD (EC 3.5.2.6) (Cysteine-rich protein D) (Penicillin-binding protein 4) (PBP 4) | -1.3909972 | 2.62259894 | 1.01E-05 |
| P56008 | Small ribosomal subunit protein bS1 (30S ribosomal protein S1) | -1.3835048 | 2.60901421 | 0 |
| P66459 | Small ribosomal subunit protein bS18 (30S ribosomal protein S18) | -1.3826385 | 2.60744803 | 3.69E-06 |
| P56102 | Methionine aminopeptidase (MAP) (MetAP) (EC 3.4.11.18) (Peptidase M) | -1.3759764 | 2.59543508 | 3.66E-06 |
| O25468 | Chorismate dehydratase (EC 4.2.1.151) (Menaquinone biosynthetic enzyme MqnA) | -1.3729591 | 2.59001257 | 0.00137278 |
| O25853 | NADH-quinone oxidoreductase subunit D (EC 7.1.1.-) (NADH dehydrogenase I subunit D) (NDH-1 subunit D) | -1.371824 | 2.58797557 | 0.00443023 |
| P56084 | ATP synthase epsilon chain (ATP synthase F1 sector epsilon subunit) (F-ATPase epsilon subunit) | -1.3701293 | 2.58493732 | 2.38E-08 |
| O06914 | Fumarate reductase iron-sulfur subunit (EC 1.3.5.1) (Quinol-fumarate reductase iron-sulfur subunit) (QFR iron-sulfur subunit) | -1.3682583 | 2.58158715 | 5.15E-07 |
| O25925 | Cell shape-determining protein MreB | -1.3667889 | 2.57895911 | 0 |
| O25787 | Uncharacterized protein | -1.3644377 | 2.57475953 | 0.00010841 |
| P56001 | DNA-directed RNA polymerase subunit alpha (RNAP subunit alpha) (EC 2.7.7.6) (RNA polymerase subunit alpha) (Transcriptase subunit alpha) | -1.363596 | 2.5732578 | 0 |
| P56129 | Diaminopimelate decarboxylase (DAP decarboxylase) (DAPDC) (EC 4.1.1.20) | -1.360448 | 2.567649 | 0.00020072 |
| P66185 | Large ribosomal subunit protein bL31 (50S ribosomal protein L31) | -1.3599868 | 2.56682831 | 1.13E-11 |
| P96786 | Flagellar hook-associated protein 2 (HAP2) (Filament cap protein) (Flagellar cap protein) | -1.3559731 | 2.5596971 | 0.00014149 |
| O26052 | Lipoprotein | -1.3407875 | 2.5328954 | 1.02E-05 |
| O25663 | Serine protease (HtrA) | -1.3350212 | 2.52279189 | 0 |
| O25560 | Hydrogenase/urease maturation factor HypB (Hydrogenase/urease nickel incorporation protein HypB) | -1.3161116 | 2.48994107 | 0 |
| P56043 | Large ribosomal subunit protein uL18 (50S ribosomal protein L18) | -1.3107273 | 2.48066565 | 0 |
| P65185 | 4-hydroxy-3-methylbut-2-enyl diphosphate reductase (HMBPP reductase) (EC 1.17.7.4) | -1.305984 | 2.4725231 | 3.93E-09 |
| P56002 | Elongation factor G (EF-G) | -1.2991489 | 2.46083666 | 0 |
| O24916 | Malonyl CoA-acyl carrier protein transacylase (EC 2.3.1.39) | -1.2930146 | 2.45039546 | 1.99E-09 |
| P56013 | Small ribosomal subunit protein bS6 (30S ribosomal protein S6) | -1.2820172 | 2.43178756 | 0 |
| O25347 | Modulator of drug activity (Mda66) | -1.2785676 | 2.4259799 | 0 |
| P55975 | Elongation factor Ts (EF-Ts) | -1.2755493 | 2.42090975 | 0 |
| P56032 | Large ribosomal subunit protein uL4 (50S ribosomal protein L4) | -1.2746504 | 2.41940183 | 4.79E-14 |
| O25313 | Ferredoxin oxidoreductase, gamma subunit | -1.2738707 | 2.41809462 | 1.12E-12 |
| O25736 | Pyruvate ferredoxin oxidoreductase, gamma subunit | -1.2689526 | 2.40986545 | 0 |
| O25144 | Zinc-metallo protease (YJR117W) | -1.2670859 | 2.40674935 | 0.00599351 |
| O25017 | Disulfide isomerase DsbG N-terminal domain-containing protein | -1.2642967 | 2.40210081 | 0 |
| P56162 | Orotate phosphoribosyltransferase (OPRT) (OPRTase) (EC 2.4.2.10) | -1.2621186 | 2.39847699 | 0.00018727 |
| O24991 | UDP-3-O-acylglucosamine N-acyltransferase (EC 2.3.1.191) | -1.2600509 | 2.39504191 | 4.01E-08 |
| P71403 | Chemotaxis protein CheY1 | -1.2458159 | 2.37152635 | 8.52E-13 |
| O25856 | NADH-ubiquinone oxidoreductase, NQO3 subunit (NQO3) | -1.2435363 | 2.36778207 | 0 |
| O24956 | Cytochrome c oxidase, monoheme subunit, membrane-bound (FixO) | -1.2422766 | 2.36571552 | 1.81E-11 |
| O24915 | Aminodeoxyfutalosine nucleosidase (AFL nucleosidase) (Aminofutalosine nucleosidase) (EC 3.2.2.30) (5'-methylthioadenosine/S-adenosylhomocysteine nucleosidase) (MTA/SAH nucleosidase) (MTAN) (EC 3.2.2.9) (6-amino-6-deoxyfutalosine N-ribosylhydrolase) | -1.2400356 | 2.36204361 | 6.20E-06 |
| O24994 | Beta-ketoacyl-[acyl-carrier-protein] synthase III (Beta-ketoacyl-ACP synthase III) (KAS III) (EC 2.3.1.180) (3-oxoacyl-[acyl-carrier-protein] synthase 3) (3-oxoacyl-[acyl-carrier-protein] synthase III) | -1.2396539 | 2.36141875 | 0 |
| O25656 | Zinc protease PqqE (EC 3.4.24.-) | -1.2394123 | 2.36102333 | 1.73E-10 |
| O25739 | Pyruvate ferredoxin oxidoreductase, beta subunit | -1.2373627 | 2.35767147 | 4.79E-06 |
| O25130 | UDP-4-amino-4,6-dideoxy-N-acetyl-beta-L-altrosamine transaminase (EC 2.6.1.92) (Pseudaminic acid biosynthesis protein C) | -1.2368155 | 2.3567774 | 4.54E-09 |
| P55980 | Cytotoxicity-associated immunodominant antigen (120 kDa protein) (CAG pathogenicity island protein 26) | -1.2338773 | 2.35198246 | 0 |
| O25742 | beta-lactamase (EC 3.5.2.6) | -1.229209 | 2.34438417 | 0 |
| P48285 | Enolase (EC 4.2.1.11) (2-phospho-D-glycerate hydro-lyase) (2-phosphoglycerate dehydratase) | -1.2280698 | 2.3425337 | 0 |
| O25265 | Cag pathogenicity island protein (Cag10) | -1.2197778 | 2.32910842 | 9.01E-05 |
| O24929 | Methyl-accepting chemotaxis protein (TlpB) | -1.2196244 | 2.32886078 | 0 |
| O25018 | UPF0323 lipoprotein HP_0232 | -1.2053779 | 2.30597664 | 0 |
| O25249 | Plasminogen-binding protein PgbA | -1.202098 | 2.30074007 | 4.08E-06 |
| O24937 | DUF874 family protein | -1.1866433 | 2.2762252 | 2.40E-05 |
| P55982 | Ribonucleoside-diphosphate reductase subunit alpha (EC 1.17.4.1) (Ribonucleotide reductase) | -1.1858206 | 2.27492755 | 4.35E-08 |
| O06912 | Fumarate reductase cytochrome b subunit (Quinol-fumarate reductase cytochrome b subunit) (QFR cytochrome b subunit) | -1.1729812 | 2.25477144 | 0.00620393 |
| P56140 | Porphobilinogen deaminase (PBG) (EC 2.5.1.61) (Hydroxymethylbilane synthase) (HMBS) (Pre-uroporphyrinogen synthase) | -1.1725393 | 2.25408091 | 0.00090884 |
| O25225 | Large ribosomal subunit assembly factor BipA (EC 3.6.5.-) (50S ribosomal subunit assembly factor BipA) (GTP-binding protein BipA) | -1.1663251 | 2.24439266 | 0 |
| P56153 | Inorganic pyrophosphatase (EC 3.6.1.1) (Pyrophosphate phospho-hydrolase) (PPase) | -1.1659803 | 2.24385632 | 0 |
| P56004 | Elongation factor P (EF-P) | -1.1504961 | 2.21990217 | 0 |
| O24983 | CvpA family protein | -1.1481584 | 2.21630802 | 3.72E-06 |
| O25345 | Beta-lactamase (EC 3.5.2.6) | -1.1470618 | 2.21462403 | 2.12E-05 |
| P55988 | ATP synthase subunit beta (EC 7.1.2.2) (ATP synthase F1 sector subunit beta) (F-ATPase subunit beta) | -1.1419178 | 2.20674174 | 0 |
| P69996 | Urease subunit beta (EC 3.5.1.5) (Urea amidohydrolase subunit beta) | -1.1394445 | 2.20296183 | 0 |
| O25840 | Outer membrane protein (Omp28) | -1.1362879 | 2.19814705 | 0 |
| O25728 | Putative beta-lactamase HcpC (EC 3.5.2.6) (Cysteine-rich protein C) | -1.1323101 | 2.19209466 | 1.34E-10 |
| O25893 | tRNA-specific 2-thiouridylase MnmA (EC 2.8.1.13) | -1.1267271 | 2.183628 | 1.99E-09 |
| Q09066 | Urease accessory protein UreG | -1.1264705 | 2.18323965 | 0 |
| O24985 | SPOR domain-containing protein | -1.1262756 | 2.18294473 | 6.35E-13 |
| O26005 | Outer membrane protein (Omp31) | -1.1262236 | 2.18286605 | 1.70E-08 |
| P56420 | Trigger factor (TF) (EC 5.2.1.8) (PPIase) | -1.11971 | 2.17303287 | 0 |
| O25865 | Phosphomannomutase (AlgC)(Pseudomonas aeruginosa) | -1.1149512 | 2.16587683 | 6.99E-12 |
| O25594 | Amino acid ABC transporter, periplasmic binding protein (YckK) | -1.1134437 | 2.16361484 | 9.00E-07 |
| O25134 | Biotin carboxylase (EC 6.3.4.14) (Acetyl-coenzyme A carboxylase biotin carboxylase subunit A) | -1.1115821 | 2.1608248 | 0 |
| O25079 | CN hydrolase domain-containing protein | -1.1028181 | 2.14773813 | 0.00944791 |
| O25277 | Cag pathogenicity island protein (Cag24) | -1.0985964 | 2.14146248 | 6.33E-08 |
| P64275 | Transcription elongation factor GreA (Transcript cleavage factor GreA) | -1.0964035 | 2.13820992 | 1.12E-07 |
| O25158 | D-3-phosphoglycerate dehydrogenase (EC 1.1.1.95) | -1.0911136 | 2.13038415 | 0 |
| O25047 | HP0268 domain-containing protein | -1.0871711 | 2.12457033 | 3.07E-06 |
| P56112 | Putative peptidyl-prolyl cis-trans isomerase HP_0175 (PPIase HP_0175) (EC 5.2.1.8) (Rotamase HP_0175) | -1.0838476 | 2.11968164 | 0 |
| O25776 | Flavodoxin | -1.0802181 | 2.1143557 | 9.50E-13 |
| O25363 | UTP--glucose-1-phosphate uridylyltransferase (EC 2.7.7.9) (UDP-glucose pyrophosphorylase) | -1.0774698 | 2.11033173 | 1.91E-07 |
| O26084 | Lipoprotein | -1.0773771 | 2.11019614 | 0 |
| O25905 | Protease | -1.0768493 | 2.10942428 | 0 |
| P56103 | Guanylate kinase (EC 2.7.4.8) (GMP kinase) | -1.0751809 | 2.10698625 | 0.00104568 |
| O25791 | Outer membrane protein (Omp27) | -1.0702074 | 2.0997352 | 0 |
| O25087 | Heme oxygenase HugZ (EC 1.14.99.-) | -1.0687 | 2.09754244 | 5.83E-15 |
| O25349 | Quinone-reactive Ni/Fe hydrogenase, large subunit (HydB) | -1.0683379 | 2.09701604 | 0 |
| O25751 | Tol-Pal system protein TolB | -1.0662956 | 2.09404958 | 6.03E-10 |
| O25030 | Band 7 domain-containing protein | -1.0654315 | 2.09279573 | 0 |
| O24943 | DUF1104 domain-containing protein | -1.0640061 | 2.09072904 | 0 |
| P55987 | ATP synthase subunit alpha (EC 7.1.2.2) (ATP synthase F1 sector subunit alpha) (F-ATPase subunit alpha) | -1.0627962 | 2.08897641 | 0 |
| P56137 | Adenylosuccinate synthetase (AMPSase) (AdSS) (EC 6.3.4.4) (IMP--aspartate ligase) | -1.0575694 | 2.08142186 | 4.11E-05 |
| O24999 | Iron-sulfur cluster carrier protein | -1.0549581 | 2.07765786 | 1.71E-08 |
| P56114 | Glutamyl-tRNA(Gln) amidotransferase subunit A (Glu-ADT subunit A) (EC 6.3.5.7) | -1.0542782 | 2.07667896 | 1.23E-14 |
| P56086 | ATP synthase subunit b (ATP synthase F(0) sector subunit b) (ATPase subunit I) (F-type ATPase subunit b) (F-ATPase subunit b) | -1.0511907 | 2.07223942 | 1.08E-07 |
| O25995 | Penicillin-binding protein activator LpoB | -1.0489563 | 2.06903249 | 0 |
| P56075 | Nucleoside diphosphate kinase (NDK) (NDP kinase) (EC 2.7.4.6) (Nucleoside-2-P kinase) | -1.0420714 | 2.05918207 | 0 |
| O25369 | Outer membrane protein assembly factor BamA | -1.041622 | 2.05854074 | 0.00871879 |
| P94851 | 36 kDa antigen | -1.039379 | 2.05534275 | 2.83E-10 |
| O25570 | Outer membrane protein (Omp20) | -1.0385466 | 2.05415721 | 1.71E-15 |
| P55994 | Chaperone protein DnaK (HSP70) (Heat shock 70 kDa protein) (Heat shock protein 70) | -1.0371007 | 2.05209952 | 0 |
| O25926 | ATP-dependent Clp protease ATP-binding subunit ClpX | -1.0306234 | 2.04290682 | 0 |
| P64101 | Putative biopolymer transport protein ExbD-like 2 | -1.0303473 | 2.04251589 | 0 |
| O25607 | Uncharacterized protein | -1.0284824 | 2.03987733 | 0 |
| P56097 | Cell division protein FtsZ | -1.0174871 | 2.02438979 | 2.30E-06 |
| O24973 | Transcriptional regulatory protein ArsR | -1.0158106 | 2.02203869 | 0 |
| O25902 | Glyceraldehyde-3-phosphate dehydrogenase (Gap) | -1.0090591 | 2.01259809 | 0 |
| P56089 | Serine hydroxymethyltransferase (SHMT) (Serine methylase) (EC 2.1.2.1) | -1.0071996 | 2.01000571 | 0 |
| O25658 | 7-alpha-hydroxysteroid dehydrogenase (HdhA) | -1.0038422 | 2.00533352 | 1.71E-15 |
| O26025 | Conserved hypothetical nifU-like protein | -1.0023392 | 2.00324545 | 2.12E-05 |
| Increased abundance in PBT2 treated cells at T12 Sub-inhibitory MIC concentration (0.625 mg/L) | | | | |
| O25973 | Histidine and glutamine-rich protein | 1.97869159 | 3.9413547 | 3.13E-12 |
| O25648 | Lipoprotein | 1.50368461 | 2.8356601 | 7.55E-08 |
| O25904 | SH3 domain-containing protein | 1.42564729 | 2.68635001 | 9.20E-08 |
| O25273 | Cag pathogenicity island protein (Cag19) | 1.19791742 | 2.29408272 | 0.000606 |
| O25341 | Solute-binding signature and mitochondrial signature protein (AspB) | 1.18763065 | 2.27778353 | 0.02200231 |
| Decreased abundance in PBT2 treated cells at T12 Sub-inhibitory MIC concentration (0.625 mg/L) | | | | |
| P56021 | Small ribosomal subunit protein uS14 (30S ribosomal protein S14 type Z) | -2.9433708 | 7.69206414 | 3.57E-09 |
| O25737 | Pyruvate ferredoxin oxidoreductase, delta subunit | -2.7905754 | 6.91905675 | 9.00E-09 |
| P56049 | Large ribosomal subunit protein uL24 (50S ribosomal protein L24) | -2.4569837 | 5.4906758 | 1.79E-05 |
| P56157 | DNA polymerase III subunit alpha (EC 2.7.7.7) | -2.2906717 | 4.89283876 | 9.75E-05 |
| P56138 | tRNA uridine 5-carboxymethylaminomethyl modification enzyme MnmG (Glucose-inhibited division protein A) | -2.141228 | 4.41137375 | 2.47E-09 |
| O25983 | Translational regulator CsrA | -2.0972326 | 4.27887813 | 0.02875259 |
| P56046 | Large ribosomal subunit protein bL21 (50S ribosomal protein L21) | -1.9128591 | 3.76554598 | 0 |
| O25088 | Sec-independent protein translocase protein TatA | -1.9030102 | 3.73992713 | 0 |
| P66637 | Small ribosomal subunit protein uS9 (30S ribosomal protein S9) | -1.9014059 | 3.73577063 | 0 |
| P56042 | Large ribosomal subunit protein bL17 (50S ribosomal protein L17) | -1.8294752 | 3.55407776 | 0 |
| P56020 | Small ribosomal subunit protein uS13 (30S ribosomal protein S13) | -1.8087514 | 3.50338958 | 0 |
| P56040 | Large ribosomal subunit protein uL15 (50S ribosomal protein L15) | -1.7949101 | 3.46993849 | 0 |
| P52093 | Bacterial non-heme ferritin (EC 1.16.3.2) | -1.7915016 | 3.46175024 | 1.90E-15 |
| P0A0X4 | Small ribosomal subunit protein uS12 (30S ribosomal protein S12) | -1.7849198 | 3.445993 | 0 |
| P55989 | Copper-transporting ATPase (EC 7.2.2.9) | -1.7617358 | 3.3910587 | 0.01039721 |
| P56162 | Orotate phosphoribosyltransferase (OPRT) (OPRTase) (EC 2.4.2.10) | -1.7591372 | 3.3849563 | 2.39E-13 |
| O25581 | Probable tautomerase HP_0924 (EC 5.3.2.-) | -1.7552253 | 3.37579023 | 7.49E-11 |
| O25135 | Biotin carboxyl carrier protein of acetyl-CoA carboxylase | -1.749462 | 3.36233161 | 9.50E-13 |
| O26042 | Iron-regulated outer membrane protein (FrpB) | -1.7134361 | 3.27940956 | 0 |
| P55976 | Transcription termination/antitermination protein NusG | -1.7068035 | 3.26436761 | 0 |
| P56464 | Acyl carrier protein (ACP) | -1.705346 | 3.26107123 | 1.95E-07 |
| O24950 | Conserved hypothetical iron-sulfur protein | -1.6650691 | 3.17128845 | 4.60E-12 |
| O25376 | Oxygen-independent coproporphyrinogen III oxidase (CPO) (EC 1.3.98.3) (Coproporphyrinogen III dehydrogenase) (CPDH) | -1.6483125 | 3.13466775 | 9.66E-07 |
| O26032 | Copper resistance protein CopD | -1.6476021 | 3.13312447 | 8.35E-05 |
| O25825 | Cytochrome c-553 (Cytochrome c553) | -1.625368 | 3.08520851 | 1.18E-12 |
| O25286 | 3-oxoacyl-[acyl-carrier-protein] reductase (EC 1.1.1.100) | -1.6056069 | 3.04323749 | 0 |
| O25308 | DUF3971 domain-containing protein | -1.5820365 | 2.99392182 | 1.10E-05 |
| P56036 | Large ribosomal subunit protein uL10 (50S ribosomal protein L10) | -1.5808422 | 2.99144421 | 0 |
| P66142 | Large ribosomal subunit protein bL28 (50S ribosomal protein L28) | -1.5686975 | 2.96636779 | 0 |
| O25873 | Conserved hypothetical secreted protein | -1.558148 | 2.94475576 | 0 |
| P55973 | Translation initiation factor IF-3 | -1.5522218 | 2.93268425 | 1.13E-09 |
| O25015 | Outer membrane protein (Omp6) | -1.533829 | 2.89553315 | 4.81E-08 |
| P56009 | Small ribosomal subunit protein uS2 (30S ribosomal protein S2) | -1.5275674 | 2.88299308 | 0 |
| P56029 | Large ribosomal subunit protein uL1 (50S ribosomal protein L1) | -1.5187906 | 2.86550736 | 0 |
| O24968 | Putative beta-lactamase HcpD (EC 3.5.2.6) (Cysteine-rich protein D) (Penicillin-binding protein 4) (PBP 4) | -1.5065611 | 2.84131959 | 1.68E-05 |
| O24959 | DUF4006 family protein | -1.4966301 | 2.82182818 | 1.53E-06 |
| P56041 | Large ribosomal subunit protein uL16 (50S ribosomal protein L16) | -1.4892664 | 2.80746182 | 0 |
| P56034 | Large ribosomal subunit protein uL6 (50S ribosomal protein L6) | -1.4753495 | 2.78050989 | 0 |
| P56030 | Large ribosomal subunit protein uL2 (50S ribosomal protein L2) | -1.4554615 | 2.7424428 | 0 |
| P56052 | Large ribosomal subunit protein uL29 (50S ribosomal protein L29) | -1.4407455 | 2.71461108 | 0 |
| O25761 | Protein CR006 P-loop domain-containing protein | -1.4404386 | 2.71403354 | 1.36E-12 |
| O25769 | Flagellar assembly factor FliW 1 | -1.4353042 | 2.70439191 | 1.13E-05 |
| O24951 | Conserved hypothetical secreted protein | -1.433934 | 2.70182462 | 1.72E-13 |
| P56010 | Small ribosomal subunit protein uS3 (30S ribosomal protein S3) | -1.4251282 | 2.68538355 | 0 |
| O25444 | Cell division protein (FtsE) | -1.4240353 | 2.68335008 | 2.23E-06 |
| P56078 | Large ribosomal subunit protein bL25 (50S ribosomal protein L25) (General stress protein CTC) | -1.4059364 | 2.64989716 | 0 |
| P66185 | Large ribosomal subunit protein bL31 (50S ribosomal protein L31) | -1.3830319 | 2.60815921 | 0 |
| P56044 | Large ribosomal subunit protein bL19 (50S ribosomal protein L19) | -1.376443 | 2.59627466 | 0 |
| P66621 | Small ribosomal subunit protein uS8 (30S ribosomal protein S8) | -1.3685412 | 2.58209347 | 0 |
| P56047 | Large ribosomal subunit protein uL22 (50S ribosomal protein L22) | -1.3670748 | 2.57947025 | 0 |
| O25898 | Biopolymer transport protein ExbD | -1.3571563 | 2.56179725 | 0.00025659 |
| O25856 | NADH-ubiquinone oxidoreductase, NQO3 subunit (NQO3) | -1.3460802 | 2.54220474 | 0 |
| O25531 | D-glycero-beta-D-manno-heptose-1,7-bisphosphate 7-phosphatase (EC 3.1.3.82) (D,D-heptose 1,7-bisphosphate phosphatase) (HBP phosphatase) | -1.3396491 | 2.53089761 | 1.93E-09 |
| P56045 | Large ribosomal subunit protein bL20 (50S ribosomal protein L20) | -1.336189 | 2.52483476 | 0 |
| P56466 | Transcription termination factor Rho (EC 3.6.4.-) (ATP-dependent helicase Rho) | -1.3359815 | 2.52447175 | 0 |
| P66609 | Small ribosomal subunit protein uS7 (30S ribosomal protein S7) | -1.3274766 | 2.5096333 | 0 |
| P66449 | Small ribosomal subunit protein uS17 (30S ribosomal protein S17) | -1.3265527 | 2.50802663 | 0 |
| P56039 | Large ribosomal subunit protein uL14 (50S ribosomal protein L14) | -1.3225789 | 2.50112803 | 5.83E-11 |
| O25663 | Serine protease (HtrA) | -1.3133736 | 2.48521999 | 0 |
| P66572 | Small ribosomal subunit protein uS5 (30S ribosomal protein S5) | -1.3124755 | 2.48367347 | 0 |
| P55834 | Large ribosomal subunit protein bL12 (50S ribosomal protein L7/L12) | -1.3114977 | 2.48199065 | 0 |
| P56038 | Large ribosomal subunit protein uL13 (50S ribosomal protein L13) | -1.3099195 | 2.47927699 | 0 |
| P56031 | Large ribosomal subunit protein uL3 (50S ribosomal protein L3) | -1.3053086 | 2.47136591 | 0 |
| O25742 | beta-lactamase (EC 3.5.2.6) | -1.3023105 | 2.46623542 | 0 |
| O26052 | Lipoprotein | -1.2893902 | 2.44424728 | 3.69E-15 |
| O26039 | Glycerol-3-phosphate acyltransferase (Acyl-PO4 G3P acyltransferase) (Acyl-phosphate--glycerol-3-phosphate acyltransferase) (G3P acyltransferase) (GPAT) (EC 2.3.1.275) (Lysophosphatidic acid synthase) (LPA synthase) | -1.2862811 | 2.4389853 | 0.01227947 |
| O25047 | HP0268 domain-containing protein | -1.2617885 | 2.39792828 | 7.23E-15 |
| O25550 | DUF342 domain-containing protein | -1.2614607 | 2.39738342 | 0.003749 |
| O06914 | Fumarate reductase iron-sulfur subunit (EC 1.3.5.1) (Quinol-fumarate reductase iron-sulfur subunit) (QFR iron-sulfur subunit) | -1.2514706 | 2.38083985 | 1.52E-13 |
| P56113 | tRNA hydroxylation protein P (EC 3.4.-.-) | -1.2502529 | 2.37883125 | 4.58E-05 |
| O24916 | Malonyl CoA-acyl carrier protein transacylase (EC 2.3.1.39) | -1.2397838 | 2.36163132 | 8.77E-09 |
| P56071 | Threonine--tRNA ligase (EC 6.1.1.3) (Threonyl-tRNA synthetase) (ThrRS) | -1.2332589 | 2.35097446 | 2.86E-09 |
| O24944 | Sodium:calcium antiporter | -1.2191453 | 2.3280875 | 0 |
| O25142 | Alpha-(1,3)-fucosyltransferase (EC 2.4.1.152) (4-galactosyl-N-acetylglucosaminide 3-alpha-L-fucosyltransferase) | -1.2075777 | 2.3094954 | 3.88E-07 |
| O25018 | UPF0323 lipoprotein HP_0232 | -1.1967145 | 2.29217074 | 5.46E-15 |
| P56001 | DNA-directed RNA polymerase subunit alpha (RNAP subunit alpha) (EC 2.7.7.6) (RNA polymerase subunit alpha) (Transcriptase subunit alpha) | -1.1943025 | 2.28834163 | 0 |
| O25759 | SpoOJ regulator (Soj) | -1.1906356 | 2.28253277 | 6.24E-08 |
| P56463 | Purine nucleoside phosphorylase DeoD-type (PNP) (EC 2.4.2.1) | -1.1850324 | 2.27368493 | 0.00010198 |
| O25009 | Nitrogen fixation protein NifU | -1.1849339 | 2.27352981 | 0 |
| O25879 | Protein translocase subunit SecY | -1.1773459 | 2.26160331 | 5.30E-06 |
| P56033 | Large ribosomal subunit protein uL5 (50S ribosomal protein L5) | -1.1772101 | 2.26139049 | 0 |
| O25065 | Phosphatidylglycerol lysyltransferase C-terminal domain-containing protein | -1.1756041 | 2.25887445 | 0.00088598 |
| P56026 | Small ribosomal subunit protein uS19 (30S ribosomal protein S19) | -1.1718136 | 2.25294741 | 5.99E-14 |
| P56032 | Large ribosomal subunit protein uL4 (50S ribosomal protein L4) | -1.1691797 | 2.24883795 | 0 |
| O26031 | Outer membrane protein (Omp32) | -1.1669495 | 2.24536432 | 2.51E-06 |
| P56008 | Small ribosomal subunit protein bS1 (30S ribosomal protein S1) | -1.1572182 | 2.23026975 | 0 |
| P66246 | Large ribosomal subunit protein bL34 (50S ribosomal protein L34) | -1.1488118 | 2.21731207 | 1.32E-06 |
| P56420 | Trigger factor (TF) (EC 5.2.1.8) (PPIase) | -1.1474813 | 2.21526801 | 0 |
| P66328 | Small ribosomal subunit protein uS10 (30S ribosomal protein S10) | -1.1461726 | 2.21325948 | 0 |
| O25536 | Deoxyuridine 5'-triphosphate nucleotidohydrolase (dUTPase) (EC 3.6.1.23) (dUTP pyrophosphatase) | -1.1443887 | 2.21052452 | 2.92E-11 |
| O26073 | Protein translocase subunit SecF | -1.1416122 | 2.20627442 | 1.96E-06 |
| O25656 | Zinc protease PqqE (EC 3.4.24.-) | -1.1311177 | 2.19028368 | 1.16E-10 |
| O25566 | Flagellar hook (FlgE) | -1.1266367 | 2.1834911 | 0.02168254 |
| P56002 | Elongation factor G (EF-G) | -1.1250645 | 2.18111305 | 0 |
| P66052 | Large ribosomal subunit protein uL11 (50S ribosomal protein L11) | -1.1249059 | 2.18087322 | 0 |
| P56011 | Small ribosomal subunit protein uS4 (30S ribosomal protein S4) | -1.1202005 | 2.17377177 | 0 |
| O25371 | Non-peptidase homolog YmxG | -1.1198816 | 2.17329142 | 0 |
| P55988 | ATP synthase subunit beta (EC 7.1.2.2) (ATP synthase F1 sector subunit beta) (F-ATPase subunit beta) | -1.1157554 | 2.16708451 | 0 |
| O25936 | Fructose-1,6-bisphosphatase class 1 (FBPase class 1) (EC 3.1.3.11) (D-fructose-1,6-bisphosphate 1-phosphohydrolase class 1) | -1.1025655 | 2.14736217 | 0 |
| O25087 | Heme oxygenase HugZ (EC 1.14.99.-) | -1.1025559 | 2.14734781 | 0 |
| P96786 | Flagellar hook-associated protein 2 (HAP2) (Filament cap protein) (Flagellar cap protein) | -1.1022869 | 2.1469475 | 1.55E-07 |
| P55980 | Cytotoxicity-associated immunodominant antigen (120 kDa protein) (CAG pathogenicity island protein 26) | -1.1017901 | 2.14620832 | 0 |
| P56142 | Tryptophan synthase beta chain (EC 4.2.1.20) | -1.0949431 | 2.13604656 | 3.92E-05 |
| O25408 | Transcriptional regulatory protein FlgR | -1.093368 | 2.13371578 | 1.27E-05 |
| P56137 | Adenylosuccinate synthetase (AMPSase) (AdSS) (EC 6.3.4.4) (IMP--aspartate ligase) | -1.0835454 | 2.11923768 | 4.49E-10 |
| P56050 | Large ribosomal subunit protein bL27 (50S ribosomal protein L27) | -1.0823608 | 2.1174983 | 9.04E-09 |
| O24937 | DUF874 family protein | -1.082257 | 2.11734589 | 7.78E-08 |
| P71403 | Chemotaxis protein CheY1 | -1.0733136 | 2.10426093 | 0 |
| P56185 | Ribonuclease J (RNase J) (EC 3.1.-.-) | -1.0708001 | 2.10059798 | 5.82E-10 |
| O25372 | Aspartyl/glutamyl-tRNA(Asn/Gln) amidotransferase subunit B (Asp/Glu-ADT subunit B) (EC 6.3.5.-) | -1.0627432 | 2.08889974 | 0 |
| O25300 | Signal peptidase I (SPase I) (EC 3.4.21.89) (Leader peptidase I) | -1.0591571 | 2.08371375 | 8.92E-05 |
| P66119 | Large ribosomal subunit protein uL23 (50S ribosomal protein L23) | -1.057843 | 2.08181668 | 0 |
| O26002 | Conserved hypothetical integral membrane protein | -1.0577532 | 2.08168712 | 0.00686933 |
| O25674 | Flagellar motor switch protein FliN | -1.0554659 | 2.07838928 | 1.77E-05 |
| O25021 | Putative beta-lactamase HcpE (EC 3.5.2.6) (Cysteine-rich protein E) | -1.0539111 | 2.07615053 | 1.97E-05 |
| O25853 | NADH-quinone oxidoreductase subunit D (EC 7.1.1.-) (NADH dehydrogenase I subunit D) (NDH-1 subunit D) | -1.0476161 | 2.06711132 | 1.63E-05 |
| O25249 | Plasminogen-binding protein PgbA | -1.0390651 | 2.05489561 | 1.28E-09 |
| O25841 | Single-stranded DNA-binding protein (SSB) | -1.0387819 | 2.05449224 | 0 |
| P55999 | Peptide chain release factor 2 (RF-2) | -1.0348824 | 2.04894664 | 1.03E-07 |
| O25008 | Cysteine desulfurase IscS (EC 2.8.1.7) | -1.0309974 | 2.0434365 | 0 |
| O25017 | Disulfide isomerase DsbG N-terminal domain-containing protein | -1.0278564 | 2.03899245 | 0 |
| P55975 | Elongation factor Ts (EF-Ts) | -1.0260072 | 2.03638055 | 0 |
| O25720 | Transketolase (EC 2.2.1.1) | -1.0224085 | 2.03130731 | 0.0006695 |
| O25006 | YbhB/YbcL family Raf kinase inhibitor-like protein | -1.0177639 | 2.02477829 | 2.05E-09 |
| P55970 | Protein GrpE (HSP-70 cofactor) | -1.0106125 | 2.01476634 | 1.20E-07 |
| O25068 | Flagellin B homolog (Fla) | -1.0098906 | 2.01375841 | 0.00383349 |
| O25030 | Band 7 domain-containing protein | -1.0074182 | 2.01031027 | 0 |
| Increased abundance in PBT2 treated cells at T12 compared to T10 Sub-inhibitory MIC concentration (0.625 mg/L) | | | | |
| O25140 | Thiol:disulfide interchange protein (DsbC), putative | 2.82294387 | 7.07604819 | 7.61E-06 |
| O25354 | Lipoprotein | 1.70552705 | 3.2614806 | 0.00365543 |
| P64275 | Transcription elongation factor GreA (Transcript cleavage factor GreA) | 1.63717629 | 3.11056421 | 5.23E-05 |
| O24915 | Aminodeoxyfutalosine nucleosidase (AFL nucleosidase) (Aminofutalosine nucleosidase) (EC 3.2.2.30) (5'-methylthioadenosine/S-adenosylhomocysteine nucleosidase) (MTA/SAH nucleosidase) (MTAN) (EC 3.2.2.9) (6-amino-6-deoxyfutalosine N-ribosylhydrolase) | 1.47241719 | 2.77486424 | 0.00043507 |
| P94842 | Acyl-CoA thioesterase YbgC (EC 3.1.2.-) | 1.42417203 | 2.68360443 | 0.00045005 |
| Q09065 | Urease accessory protein UreF | 1.35590561 | 2.55957735 | 4.19E-09 |
| P56108 | Transaldolase (EC 2.2.1.2) | 1.32399132 | 2.50357785 | 0.02691648 |
| P56028 | Small ribosomal subunit protein bS21 (30S ribosomal protein S21) | 1.30627759 | 2.47302631 | 0.01145094 |
| O26031 | Outer membrane protein (Omp32) | 1.28705429 | 2.44029286 | 3.32E-06 |
| O25028 | Flagellar P-ring protein (Basal body P-ring protein) | 1.23020122 | 2.34599708 | 0.00012895 |
| P94845 | Glutamine synthetase (GS) (EC 6.3.1.2) (Glutamate--ammonia ligase) (Glutamine synthetase I beta) (GSI beta) | 1.22992847 | 2.3455536 | 7.69E-06 |
| O25785 | Probable ABC transporter ATP-binding protein PEB1C | 1.13701988 | 2.19926261 | 1.74E-06 |
| O25586 | Uncharacterized protein | 1.12824485 | 2.18592644 | 0.00040704 |
| P56129 | Diaminopimelate decarboxylase (DAP decarboxylase) (DAPDC) (EC 4.1.1.20) | 1.12159757 | 2.17587785 | 0.00092617 |
| P55977 | Transcription termination/antitermination protein NusA | 1.08066016 | 2.11500367 | 0.02373362 |
| O25360 | GlutamylGlutaminyl-tRNA synthetase (GluGlnRS) (EC 6.1.1.-) (Glutamate--tRNA ligase 2) (GluRS 2) (Glutamyl-tRNA synthetase 2) | 1.06742143 | 2.09568434 | 0.035691 |
| O24884 | mannose-1-phosphate guanylyltransferase (EC 2.7.7.13) | 1.06647153 | 2.09430495 | 0.00078233 |
| O25468 | Chorismate dehydratase (EC 4.2.1.151) (Menaquinone biosynthetic enzyme MqnA) | 1.05732581 | 2.08107046 | 7.12E-05 |
| P56102 | Methionine aminopeptidase (MAP) (MetAP) (EC 3.4.11.18) (Peptidase M) | 1.02444642 | 2.03417869 | 0.00097417 |
| P25177 | Phosphoglucosamine mutase (EC 5.4.2.10) | 1.02145459 | 2.02996463 | 1.24E-05 |
